# Anodic TiO_2_ Nanotubes: Tailoring Osteoinduction via Drug Delivery

**DOI:** 10.3390/nano11092359

**Published:** 2021-09-11

**Authors:** Jung Park, Anisoara Cimpean, Alexander B. Tesler, Anca Mazare

**Affiliations:** 1 Division of Molecular Pediatrics, Department of Pediatrics, University Hospital Erlangen, 91054 Erlangen, Germany; jung.park@uk-erlangen.de; 2 Department of Biochemistry and Molecular Biology, University of Bucharest, 91-95 Spl. Independentei, 050095 Bucharest, Romania; anisoara.cimpean@bio.unibuc.ro; 3 Department of Materials Science WW4-LKO, Friedrich-Alexander University, 91058 Erlangen, Germany; alexander.tesler@fau.de

**Keywords:** anodic TiO_2_, TiO_2_ nanotubes, osteoinduction, electric field, drug delivery

## Abstract

TiO_2_ nanostructures and more specifically nanotubes have gained significant attention in biomedical applications, due to their controlled nanoscale topography in the sub-100 nm range, high surface area, chemical resistance, and biocompatibility. Here we review the crucial aspects related to morphology and properties of TiO_2_ nanotubes obtained by electrochemical anodization of titanium for the biomedical field. Following the discussion of TiO_2_ nanotopographical characterization, the advantages of anodic TiO_2_ nanotubes will be introduced, such as their high surface area controlled by the morphological parameters (diameter and length), which provides better adsorption/linkage of bioactive molecules. We further discuss the key interactions with bone-related cells including osteoblast and stem cells in in vitro cell culture conditions, thus evaluating the cell response on various nanotubular structures. In addition, the synergistic effects of electrical stimulation on cells for enhancing bone formation combining with the nanoscale environmental cues from nanotopography will be further discussed. The present review also overviews the current state of drug delivery applications using TiO_2_ nanotubes for increased osseointegration and discusses the advantages, drawbacks, and prospects of drug delivery applications via these anodic TiO_2_ nanotubes.

## 1. Introduction

Titanium (Ti) and titanium-based alloys are one of the most widely used metallic materials in biomedical applications, e.g., in implants, as they possess by far the most perfect mix of properties, including high biocompatibility and corrosion resistance, good tensile strength, as well as flexibility [1,2]. The high biocompatibility of Ti arises from its inertness and chemical resistance due to the low electrical conductivity that contributes to the electrochemical oxidation of Ti, forming a thin passive oxide barrier layer. The latter, in turn, leads to the high resistance of Ti to corrosion [1,3], as well as being responsible for its high surface energy characteristics [4].

The choice of metallic biomaterials between pure Ti or other Ti alloys depends on the targeted application, e.g., Ti or Ti6Al7Nb, Ti6Al4V, Ti13Cu4.5Ni, Ti25Pd5Cr, or the more recent TiNi, TiNiAg, TiZr for dental implants [1,2] and Ti6Al4V or more recently Ti6Al7Nb or Ti5Al 2.5Fe for orthopedic applications [2] are preferred. The recent paradigm shift in the biomedical field from the microscale to nanoscale topography [5,6] was also applied to nanostructures on Ti and its alloys [7,8,9,10]. 

Simultaneously with the shift to miniaturization of surface topography, the spotlight of the biomaterial field moved also to more complex systems based on interdisciplinary works, such as materials design, surface functionalization, nanomanufacturing of devices, and tissue engineering [11]. Among these, the modification of the surface properties of biomaterials (surface nanoscale topography, physical properties, and surface chemistry) [9,12,13] is a critical factor affecting the biocompatibility and efficiency of drug delivery and other biomedical applications [10,11,13,14,15,16,17,18,19]. Especially for biomaterials targeting dental/orthopedic bone regeneration, a good osteoinduction and optimal osseointegration with necessary mechanical properties are key aspects to consider. Recent studies showed that the surface properties of implantable biomaterials play a vital role in both (i) establishing a stable fixation of the implant and the osseointegration by preventing fibrous tissue engagement, and (ii) are accountable for local immune responses during wound healing and regeneration processes [11,16,17,20,21].

The relevant surface modification methods include so far: (a) mechanical methods (grinding, machining, etc.) [13,22], (b) acidic treatments (e.g., sulfuric or hydrochloric acid for cleaning, inducing some roughness and a more efficient deposition of additional bioactive layers) [13,23,24], (c) hydrogen peroxide [25,26], (d) hydroxyapatite coatings on Ti either by micro-arc oxidation, sol-gel methods or plasma spraying [27,28,29,30], (e) silver (Ag) coatings on Ti by plasma sputtering of other Ag containing diamond-like carbon coatings on Ti [31,32,33], (f) electrochemical anodization allowing a nanostructured layer (nanopores, nanotubes or mesosponge) to grow directly on the metallic biomaterial (Ti or Ti alloys) [9,34]. These mechanical, physical, or chemical methods enable morphological surface modification and can be combined with the addition of a coating layer on the Ti or Ti alloys surface. 

**Figure 1:**
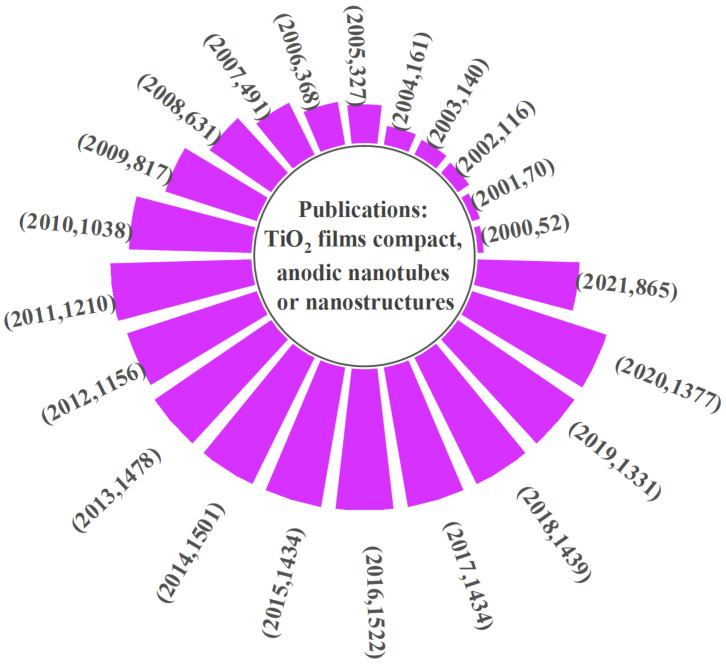
Publication record (year, publication number) in the 2000–August 2021 timeframe (Scopus–Elsevier) considering TiO_2_ compact and anodic TiO_2_ nanotubes or nanostructures.

Among the available surface modification techniques, electrochemical anodization is one of the most widely used approaches for Ti, already used for nanostructuring of the surface for a wide range of applications (photocatalytic, photoelectrochemical, batteries, biomedical, etc.). This is due to its controlled nanoscale morphology, high aspect ratio self-organized nanostructures, facile use, and good integrity of layers grown directly on the substrate [34]. Figure 1 demonstrates the growth in research and applications based on anodic titanium dioxide (TiO_2_) layers encountered in the last 20 years.

Nanostructures grown under various electrochemical anodization conditions can be further modified by adjusting the crystallinity (amorphous, annealed to anatase, or anatase/rutile) or modified with active molecules for targeted applications [35,36,37,38,39].

Surface modification of Ti or Ti alloys provides several aspects critically affecting the successful osseointegration and bone regeneration, e.g., the response of the neighboring cells to the implant (migration, adhesion, proliferation, and mineralization of cells), a foreign body reaction/osteoclastic activity, and antibacterial activity. Various methods to modify micro and/or nano-structural topography have been developed to influence cell adhesion and proliferation, [40] and osteogenic activities [41,42].

The key benefits of anodic nanotubular structures are the nanotopographical advantages that are useful in osseointegration, as well as drug delivery applications due to the high surface area of nanostructures [7,9,34]. Recently, surface modifications based on electrochemical anodization followed by further decoration by active molecules or drugs were widely introduced for improving and tailoring osseointegration accompanied with a minimal immune response [20,35,43].

For the present review, we will confine our discussion only to nanotubular structures obtained via electrochemical anodization of Ti or its alloys and their uses for drug delivery applications tailoring osteoinduction, though several remarkable nanostructure technologies for biomedical application besides anodization have been developed. The present review aims to be a clear overview of the state-of-the-art of anodic nanotubes (NTs) and their properties targeting bioapplications. At first, we will discuss the nanoscale morphology of anodic TiO_2_ nanotubes and the control over their formation. Next, the properties of nanotubes will be discussed, in particular in terms of biomedical applications, including the influence of micro- and nanoscale topography, surface roughness, and wetting behavior. We further introduce cell responses to such nanotubular substrates including growth and differentiation of bone-related cell types (mesenchymal stem cells, osteoblasts, and osteoclasts) on anodic nanotubes and electric-field induced osteogenic differentiation on TiO_2_ nanotubes. In the last section, we present recent trends of TiO_2_ nanotubes modification in drug delivery targeting osteoinduction via several application strategies to maximize the loading of anodic TiO_2_ nanotubes and to prolong the release rate or induce beneficial biological effects.

## 2. Anodic TiO_2_ Nanotubes

### 2.1. Nanomorphology and Critical Aspects of Anodic TiO_2_ Nanotubes

#### 2.1.1. Electrochemical Anodization

The shift of the materials from micro- to nanoscale topography has resulted in a large number of published works dealing with the nanostructuring of biomedical metals or alloys. In particular, nanostructuring of Ti and Ti alloys via electrochemical anodization was extensively explored. The principle of anodization and the resulting self-organization is quite simple, i.e., under optimal conditions, a steady state is achieved between oxide formation and chemical dissolution, while this balance can be influenced by several factors including anodizing potential, temperature, electrolyte composition, to name but a few [34].

**Figure 2:**
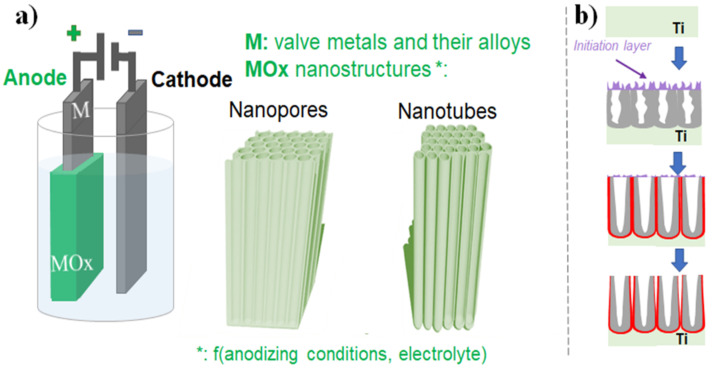
Schematic of the electrochemical anodization process: (**a**) the electrochemical anodization setup and possible resulting nanostructures, (**b**) formation of the nanotubes (red—fluoride-rich layer).

Briefly, electrochemical anodization of Ti and its alloys is performed in an electrochemical cell, with the metal/alloy of interest is used as an anode and a metal counter electrode such as Pt used as a cathode, as shown in Figure 2a. If a high enough voltage is applied and without the presence of fluoride ions in the electrolyte, the metal (M, usually valve metals such as Ti, Ta, Zr, Nb, etc., and their alloys) is oxidized (Equation (1)) and can than undergo the formation of a metal oxide ($MO_{x/2}$, Equation (2)), or is solvated followed by its dissolution in the electrolyte ($M_{solv}^{x+}$, Equation (3)) [34,44]. At the same time, at the cathode hydrogen gas is produced (Equation (4)) [34].

(1) $$M\to M^{x+}+xe^{-}$$

(2) $$M^{x+}+\frac{x}{2}H_{2}O\to MO_{x/2}+xH^{+}+xe^{-}$$

(3) $$M^{x+}+solvent\to M_{solv}^{x+})\;$$

(4) $$xH^{+}+xe^{-}\to \frac{x}{2}H_{2}$$

It should be noted that thermodynamic aspects such as oxide stability and solubility, as well as the corresponding reaction rates determine the equilibrium between oxide formation and dissolution (namely between Equations (2) and (3). However, in systems where the oxide is not soluble and no additional side reactions take place, the oxide formation dominates, leading to very high efficiencies in the formation of the oxide [34,44]. Nevertheless, an equilibrium between the film formation and dissolution can be obtained, if there is some solubility of the oxide, which will result in a steady-state situation. In this respect, crucial is that, in the presence of fluoride ions, Ti^4+^ ions can be solvatized by forming fluoro-complexes (either by chemical dissolution of the oxide—Equation (5)—or by direct complexation of the metal cations at the oxide/electrolyte interface—Equation (6)) [34,44].

(5) $$MO_{2}+6F^{-}\overset{H^{+}}{\to }{\left[MF_{6}\right]}^{2-}+2H_{2}O\;\left(M=Ti\right)$$

(6) $$M^{4+}+6F^{-}\to {\left[MF_{6}\right]}^{2-}\;\left(M=Ti\right)$$

When there is a competition between oxide formation and solvatization, where the latter is aided by the presence of fluoride ions in the case of titanium dioxide, the obtained steady-state condition results in the growth of a porous oxide; moreover, if there are optimum formation and dissolution rates, pores or nanotubes are formed [34,44]. The concentration of fluoride ions is key, as if too high, no oxide formation is observed (the controlling step of the reaction is the diffusion of the ${\left[TiF_{6}\right]}^{2-}$ complex to the surface, as the complex formation is very fast), or if to low, a stable compact oxide layer is formed [34,44]. Porous or tubular layers are obtained in an intermediate fluoride ion concentration range, where the oxide formation/Ti^4+^ solvatization competition can be easily observed. For example, in the nanotube formation process, three stages are usually present: (i) the initial state, when a compact oxide formation; (ii) the second stage, nanoscale pores are irregularly formed and penetrate the compact oxide layer formed in (i); (iii) the third stage, when regular nanotubes formation occurs. A simplified schematic of the tube growth is also shown in Figure 2b, following the above-mentioned stages, where initially a compact oxide layer is formed, followed by irregular nanopores development which grow into self-organizing nanotubular structures finally reaching a steady-state formation rate. Typical of the anodic nanopores or nanotube layer is also the formation of the fluoride-rich layer throughout the whole nanotube length (as indicated also in Figure 2b), due to the fast migration of fluoride ions through the growing oxide layer.

For additional details related to more specific mechanistic aspects and more experimental investigations regarding the formation of such specific tubular shapes, the reader is referred to the following references [34,44,45].

The nanostructure morphology depends on the anodization conditions and the electrolytes, while nanostructures such as nanopores (with no individual separation in between the pores) and nanotubes (distinct separation of the cell boundaries into individual tubes) were obtained [34,44]. Anodic nanochannels or mesoporous structures can also be obtained when anodization is performed in hot glycerol phosphate electrolytes [16,46,47]. In the following section, we will discuss morphological aspects of anodic nanotubes and how to control the tube morphology as a function of the anodization parameters.

#### 2.1.2. Morphology Aspects of Anodic TiO_2_ Nanotubes

**Figure 3:**
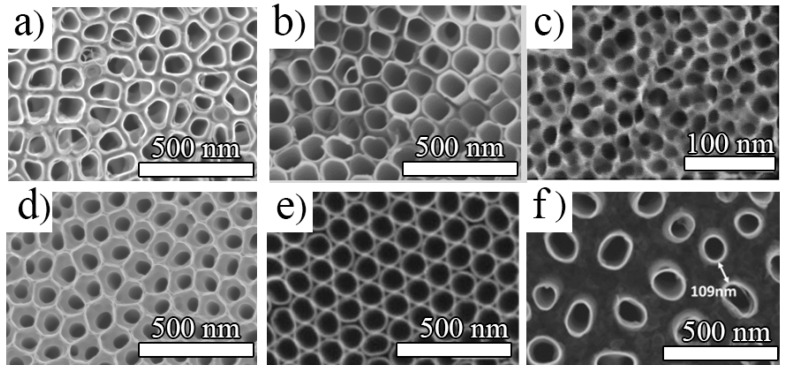
Scanning Electron Microscopy (SEM) images of different TiO_2_ nanostructures that can be grown by electrochemical anodization: (**a**) nanotubes grown in Glycerol:H_2_O (60:40 vol.%) + NH_4_F (reprinted with permission from ref. [20]. Copyright 2014 Elsevier), (**b**) open-top 100 nm diameter nanotubes grown in ethylene glycole (EG) with a low amount of water (4 M) + HF (reprinted with permission from ref. [55] Copyright 2016 Elsevier), (**c**) nanopores grown in EG with the low amount of water (6 M) + HF, (reprinted with permission from ref. [55] Copyright 2016 Elsevier), (**d**) nanotubes grown in EG, a lower amount of water (2%) + NH_4_F in a 2 step anodization approach showing a typical initiation layer, (**e**) nanotubes in o-H_3_PO_4_ + NH_4_F (reprinted with permission from ref. [56]. Copyright 2020 American Chemical Society), and (**f**) spaced nanotubes grown in diethylene glycol (DEG), low amount of water (1 wt.%) + HF (reprinted with permission from ref. [57]. Copyright 2016 Elsevier).

The different porous and tubular morphologies that can be obtained by electrochemical anodization of Ti are summarized in Figure 3. These include nanotubes obtained in glycerol: water (H_2_O) and ammonium fluoride (NH_4_F) electrolytes with a higher water content (30–50 vol.%), which show a uniform morphology (Figure 3a). The length, however, is limited to around 2 µm (see for example ref. [17,48,49]). In particular, when organic-based electrolytes with a lower amount of water (e.g., in between 1–20%) were used, nanostructures with much high aspect ratios were obtained (up to tens of µm for the lower water content electrolytes) [50,51] and can reach even faster growth rates by the use of additives (such as lactic acid) [52,53] or a more uniform morphology by surface pre-treatments (electropolishing [54], deposition of a photoresist [51]).

An open-top morphology can be obtained under specific conditions, i.e., when hydrofluoric acid (HF) is used as the fluoride source [55] (see Figure 3b), or if an optimal mild ultrasonication is performed—either in water [9], ethanol/isopropanol [58], or in water with a very low amount of HF [59]). However, tubes are commonly covered by an initiation layer, either more compact or nanograss (when using NH_4_F as fluoride source, or a double anodization approach) [60]—see also Figure 3d showing a typical initiation layer for a double anodization approach. It was previously shown that for nanotube formation in low water content electrolytes (up to 0.5 vol.% water), the nanotubes are formed from an ordered porous oxide by following a pore-wall-splitting mechanism [61], i.e., by the selective dissolution of the fluoride-rich layer that exists at the hexagonal cell boundary of the nanoporous oxide layer. This can be significantly influenced by the water content in the electrolyte as well as the fluoride amount [61,62]. See also a typical example of nanopores in Figure 3c (EG + 6 M H_2_O + 0.2 M HF, 1 h at 10 V) [63].

Very ordered, regular, and highly-uniform TiO_2_ nanotubes (initially referred to as nanostumps) can be obtained in concentrated H_3_PO_4_/NH_4_F electrolytes at high temperatures (100 °C) [64,65], while the intertube distance decreases by increasing the temperature in the 75–140 °C range [56]—such a nanotubular structure is shown in Figure 3e. More interestingly, a nanotubular morphology with a two-scale organization and a distinct space in between each nanotube from the top to the bottom of the tube length can be obtained in certain organic electrolytes, such as diethylene glycol (DEG) [57,66], triethylene glycol [67], or dimethyl sulfoxide [66,68]. As shown in Figure 3f, such nanostructures (e.g., grown in DEG-based electrolytes) are highly uniform and provide additional functionality due to the spaced morphology.

**Figure 4:**
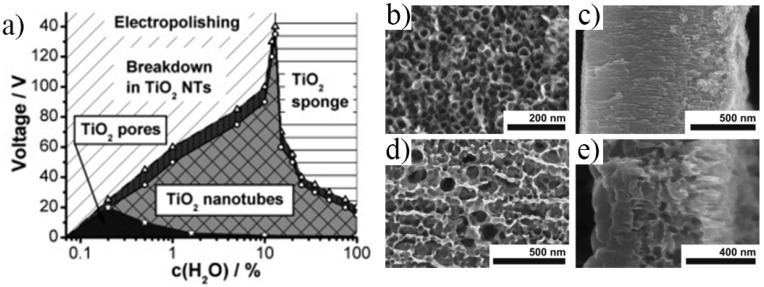
(**a**) Voltage–water concentration diagram showing the different growing regions for nanostructures (TiO_2_ nanopores, nanotubes, or sponge) for ethylene glycol-based electrolytes (reprinted with permission from ref. [69]. Copyright 2010 John Wiley and Sons). Representative SEM top and cross-section images for (**b**,**c**) nanopores—low voltage, low water content electrolyte, (**d**,**e**) sponge–high water content (**b**–**e**: reprinted with permission from ref. [69]. Copyright 2010 John Wiley and Sons).

The key factors to control the morphology of the nanostructures are well-established in the literature and can briefly be summarized as follows: the inner diameter is directly influenced by the applied voltage and the tube length by the anodization time, and both are strongly influenced by the electrolyte composition (water content and fluoride amount) [34,44,69]. A very good overview of the influence of water content on the nanostructure morphology (i.e., nanopore, nanotube, or sponge, grown in EG-based electrolytes) is shown by Albu et al. [69]—see Figure 4 for the regions of existence and representative SEM (Scanning Electron Microscopy) images of the nanopore and sponge morphologies.

Namely, if the applied voltage becomes too high, tube breakdown occurs and this for both a lower (<13 wt.%) or higher content of water, but in the case of the latter it leads to the formation of a sponge-like morphology. The water content influences the potential range into which nanopores can be grown [69]. For instance, anodization in an EG-based electrolyte with HF at 10 V, in an electrolyte with a 2–6 M H_2_O content will result in a nanopore morphology, similar to the nanopore morphology in Figure 3c. However, for the higher H_2_O content of 6 M, the pore-wall-splitting transition is shifted to much lower anodization times, e.g., of 1 h from 2.5 h, compared to lower water content electrolytes [63].

**Figure 5:**
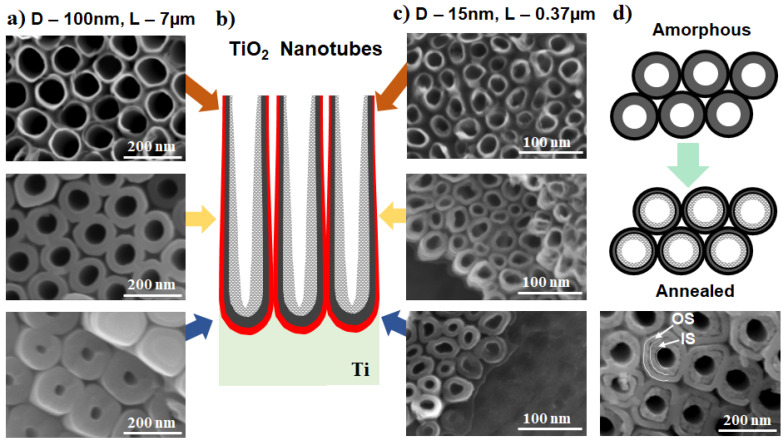
Schematic of the V-shape of nanotubes (**b**) and corresponding SEM images for nanotubes (D—diameter, L—length) with (**a**) 100 nm D nanotubes with 7 µm L (reprinted with permission from [55] Copyright 2016 Elsevier), and (**c**) 15 nm D nanotubes with 0.37 µm L (reprinted with permission from [55] Copyright 2016 Elsevier). (**d**) Separation into an inner shell (IS) and outer shell (OS) visible by annealing and a representative SEM image showing the double-wall structure.

Nowadays, to take full advantage of the high aspect ratio of anodic nanostructures, anodic nanotubular structures obtained predominantly in organic-based electrolytes are used. As a result, the “V-shape” of the nanotubes, more pronounced for nanotubes grown in organic electrolytes such as ethylene glycol (EG), has to be considered for applications where the available surface area is crucial. This shape means that the inner diameter is larger at the top and smaller at the bottom (as shown in Figure 5a–c), due to the chemical etching occurring at the top of the nanotubes. The V-shape is easier to view for larger diameter nanotubes with a high aspect ratio, but it is also present for nanotubes with diameters of 15 nm and a length of 0.37 µm. Similarly, a characteristic of the nanotubes grown in most organic-based fluoride-containing electrolytes is the double-wall structure, with an outer shell (OS) and a rich carbon inner shell (IS) [34]—as shown in Figure 5d. The latter is more easily observed after the annealing of the nanotubular layers. For many applications, the IS can be removed by decoring (i.e., a chemical etching treatment that leads to the removal of the C-rich inner layer) [70], or by using specifically designed anodization conditions where only a single-wall nanotube structure is obtained (e.g., mixed EG and dimethylsulfoxide electrolytes DMSO [71] or in DMSO electrolytes [68]).

More importantly, from the various Ti alloys used as dental or orthopedic alloys, such as Ti6Al4V, Ti6Al7Nb, Ti13Cu4.5Ni, Ti25Pd5Cr, Ti20Cr0.2Si, Ti13Nb13Zr, Ti12Mo6Zr, TiMo alloys, Ti22Nb2Cr, TiZr alloy, etc. [72,73], only some can be anodized successfully to a uniform nanostructure by electrochemical anodization. For instance, anodic nanostructures in the form of nanotubes can be grown on Ti6Al4V [74,75], Ti6Al7Nb [74,76], TiZr alloys with Zr amount in the 5–50 wt.% [77,78], Ti24Zr10Nb2Sn [79], Ti13Zr13Nb [80,81], Ti28Zr8Nb [82], TiMo alloys (6–7 wt.% Mo [83,84], 15 wt.% [84]), TiNb alloys [85,86]. Additionally, specifically designed alloys can also be anodized, such as TiNbZr/Hf (Ti25NbxZr and Ti25NbxHf with x = 0.7 and 15 wt.% alloying element [87], Ti29NbxZr with x = 3, 15 wt.% Zr [88,89], Ti35NbxZr with x = 3–10 wt.% Zr [90]), Ti35Nb5Ta7Zr [91], Ti24Nb4Zr8Sn [92], TixNb2Ag2Pt with x = 10, 30 and 50 wt.% [93], TiTa alloys [94], and other ternary alloys as Ti30TaxZr (with x = 3, 15 wt.% Zr) [89] or NiTi shape memory alloy [95,96]. Depending on the amount and type of alloying element, the anodization conditions or the tube growth range can differ to some extent in comparison to Ti anodized in the same electrolyte and under similar conditions. For more details, the reader is referred to the above-mentioned references and the dedicated reviews for anodization of biomedical alloys [97,98,99,100].

### 2.2. Key Properties and Their Improvement for Biomedical Applications

Up to this point, the different morphology of TiO_2_ nanotube layers obtained on Ti and Ti alloys by electrochemical anodization have been introduced, in the next section, an overview of the crystallinity, surface roughness, corrosion protection, and wettability will be discussed. Properties such as topography, nanoscale roughness, chemical composition, wettability, and surface charge distribution, are key in influencing and controlling the interaction of the biomaterial’s surface with osteogenic cells and early bone response enabling the implant integration, decreasing the inflammatory response, as well as the bacterial adhesion or the foreign body response [1,10,22,101,102].

#### 2.2.1. Crystallinity of Anodic TiO_2_ Nanotubes

As-anodized TiO_2_ nanotubes are amorphous; hence, to improve their properties and functionality, crystallinity is induced by thermal annealing treatments performed generally in air, at temperatures in the range of 250–750 °C, which convert the layers to an anatase or anatase/rutile crystalline structure [34,103,104,105]. However, the anatase phase starts to appear from temperatures as low as 250 °C and rutile from temperatures of around 450–500 °C (to note that the rutile growth starts from the Ti metallic interface) [34,105]. It was demonstrated that annealing TiO_2_ nanotube membranes, which were removed from the Ti substrate, resulted in an anatase crystalline phase only, at temperatures of up to 950 °C [53].

The annealing time has a key influence on the crystallinity as longer annealing time at lower temperatures also results in crystallization to anatase [103]. There is a large number of works evaluating the crystallinity of TiO_2_ nanotubes, achieved by annealing in air. In addition, further evaluation of the morphological changes occurring in the nanotubular structure due to the appearance of rutile (due to sintering) or due to morphological aspects of the double-wall structure of the nanotubes are discussed—see comprehensive reviews [34,106] and experimental works [53,103,104,105,107,108,109]. It should be noted, that in the case of mesosponge or mesoporous nanostructures obtained by anodization in hot-glycerol electrolytes the obtained nanostructures are already partially crystalline [47,110].

**Figure 6:**
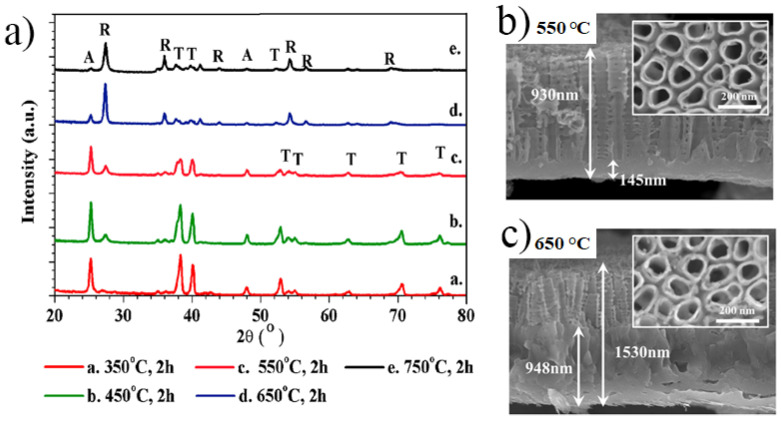
≈ 1 µm long TiO_2_ nanotubes annealed for 2 h at temperatures in the 350–750 °C range: (**a**) X-Ray diffraction (XRD) patterns and selected SEM images showing the rutile thermal oxide layer growth (reprinted with permission from ref. [111]. Copyright 2016 Elsevier), and (**b**,**c**) nanotube morphology as a function of the annealing treatment, i.e., 550 °C and 650 °C, respectively (reprinted with permission from ref. [111]. Copyright 2016 Elsevier).

As in the case of biomedical applications targeting osseointegration, the nanostructures have to be grown directly on the surface of the implant, a clear example of the X-ray diffraction (XRD) patterns of 1 µm long TiO_2_ nanotubes with 80 nm diameter annealed at temperatures up to 750 °C is shown in Figure 6. No significant differences were reported in the tube morphology for annealing up to 450 °C [111], but for higher temperatures of 550–750 °C, an increase in the rutile thermal oxide layer starting at the oxide/metal interface was observed (see also the cross-sectional SEM images in Figure 6b,c showing the thermal rutile layer). More so, for thermal treatments at temperatures higher than 650 °C, the crystallization process was accompanied by sintering (collapse) of the nanotubular layer [111].

It is worth mentioning that after thermal crystallization of the TiO_2_ nanotubes into anatase or anatase/rutile mixtures, a significant loss of water, fluoride, or carbon compounds frequently present in the nanostructures occurs (carbon comes from the inner shell of the classic double-wall nanotubes grown usually in organic electrolytes such as ethylene glycol), which also influences their wetting characteristics [34,66,71].

#### 2.2.2. Surface Roughness of Anodic TiO_2_ Nanotubes

A general trait of biomaterials is that biologically active implant materials possess increased surface roughness, which is one of the vital characteristics improving cell response to the implantable materials. A correlation between the surface roughness and osteoblast cell attachment was demonstrated [22,42], together with an effect on other aspects of a successful implant osteointegration (e.g., a selective protein adhesion, chondrocyte maturation) [112]. Nevertheless, though in vivo studies have shown that surfaces with a microstructure enable an improved contact between surface and bone and after implantation, increased mechanical retention [113,114], overall, more information is required, particularly for the recent developments in nanostructured surfaces [115].

The roughness of the biomaterial also influences additional properties such as higher local electrostatic charge density on the surface, [101] and in a combination with hydrophilicity can result in improvements in the early stages of bone healing or osteoporosis [116,117,118].

**Figure 7:**
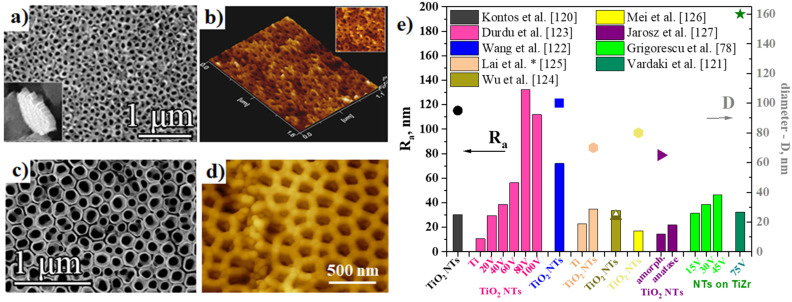
Top-view SEM image of nanotubes and corresponding 2D AFM (Atomic Force Microscopy) topography image for nanotubes grown (**a**,**b**) in an aqueous electrolyte on Ti foil (reprinted with permission from ref. [119]. Copyright 2010 Elsevier), or (**c**,**d**) in an organic electrolyte on TiZr alloy (reprinted with permission from ref. [121]. Copyright 2019 Elsevier). (**e**) Overview of Ra values on Ti, TiO_2_ nanotubes or nanotubes on TiZr alloy (Ra and diameter values from refs. [78,120,121,122,123,124,125,126,127]; *—value of Rq).

Figure 7a,b shows typical AFM (Atomic Force Microscopy) 2D topography and corresponding SEM images of NTs obtained by anodization of Ti in HF-containing aqueous electrolyte at an applied potential of 20 V for 20 min [119]. Similar NTs and AFM results were also obtained in 1 M sodium dihydrogen phosphate (NaH_2_PO_4_) + 0.3 wt.% HF, 20 V, 2 h [120], or as listed in Figure 7c,d for NTs grown on TiZr alloy by an optimized double anodization approach in a glycerol-based electrolyte with 15 vol.% H_2_O and 0.2 M NH_4_F (75 V, 1 h in the 2nd step) [121]. An overview of the average roughness (Ra) values, which is arithmetic means of the deviation in height from the profile mean value, encountered in literature for NTs grown on Ti or Ti alloys, are compiled in Figure 7e. While Ti has reported Ra values in the range of 10.7–22.7 nm, NTs have roughness values in the range of 17–112 nm depending on morphology, i.e., diameter size, where usually higher applied voltages lead to a certain point to higher diameters [78,119,120,121,122,123,124,125,126], or, to a certain extent, to the annealing treatments (a slight increase in roughness is observed, with increasing the annealing temperature and the appearance of the rutile crystalline phase) [127]. In addition, also the inner diameter value is included in this overview where available (either not mentioned in the respective article or values were in a wide range of values due to the initiation layer covering the nanotubes). Peng et al. [128] has reported much higher Ra values for both Ti and TiO_2_ NTs (i.e., 1.02 ± 0.06 µm for Ti, and 0.95 ± 0.02 µm for NTs). Based on the data presented above, there can be no conclusion concerning a possible correlation between tube diameter and average roughness; however, there is some variance in the values due to tube morphology, obtaining conditions, and top morphology of the NTs (open top, initiation layer, ultrasonication performed to remove the nanograss, etc.).

#### 2.2.3. Wetting Characteristics of Anodic TiO_2_ Nanotubes

As mentioned previously, the early bone response is influenced greatly by surface properties (e.g., topography, roughness, chemical composition, and surface energy) [1,10,22,101,102], which in turn influences the wettability of solid surfaces. Wetting is the ability of a liquid to maintain contact with a solid surface, resulting from their intermolecular interactions, while the degree of wetting is determined by a force balance between adhesive and cohesive forces called the contact angle (CA) [129]. As the tendency of a drop to spread out over a flat solid surface increases, the CA decreases. A CA less than 90°, i.e., hydrophilic surfaces, usually indicates that wetting of the surface is favorable, and the fluid will spread over a large area of the surface. In contrast, CAs greater than 90°, i.e., hydrophobic surfaces, indicating that the wetting of the surface is unfavorable, and the fluid will minimize its contact with the surface by forming a liquid droplet. There are also two extreme wettability ranges, in which the CA either exceeds 150°, namely superhydrophobic state (SHS), or goes below 10°, namely superhydrophilic state. Such extreme cases can be only achieved on rough surfaces [130]. Besides the general studies on hydrophilic/hydrophobic surfaces, there are both fundamental and practical interests to extend the investigation of the interaction between proteins or cells and surfaces to the extreme wettability ranges.

Since biomaterials used in medical devices are intended to come into intimate contact with living cells and biological fluids, their surface wettability should be prospectively designed [131]. Firstly, the coating of the implant materials with proteins from blood and interstitial fluids will occur [10,132,133]. Therefore, the regulation of wettability and protein adsorption on implanted surfaces is a key aspect in the field of regenerative biomedicine and tissue engineering.

**Figure 8:**
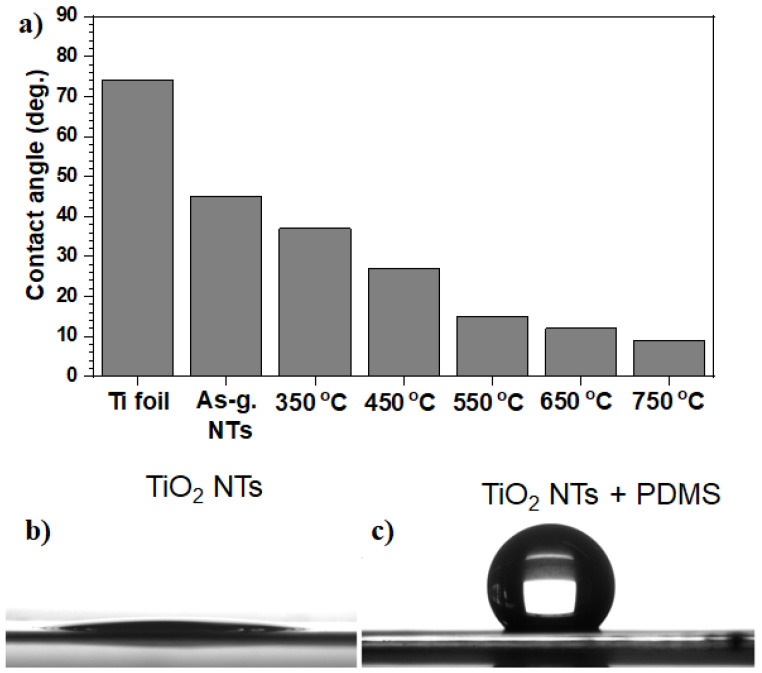
(**a**) Typical contact angle values for Ti foil, as-grown nanotubes (NTs), and NTs annealed at different temperatures in the temperature range from 350 to 750 °C (data taken from reference [111]). Typical contact angle for (**b**) bare highly ordered TiO_2_ NTs and (**c**) coated by polydimethylsiloxane (PDMS) molecules using ultraviolet (UV) light-grafting technique.

Immediately after implantation, water molecules bind to the surface forming a water mono- or bilayer. Here, their arrangement depends on the implant surface properties at the atomic level accompanied by hydrated ions, such as Cl^−^, Na^+^, and Ca^2+^, followed by blood and tissue-specific proteins that adsorb/desorb on the surface [134]. It is common knowledge nowadays that the adsorption of proteins, as well as cell adhesion on implantable surfaces, depend strongly on its structure and topography. In this sense, the electrochemical anodization of implantable Ti substrates induces the following surface modification: (i) it forms a layer of TiO_2_ material, which is intrinsically hydrophilic, and (ii) introduces micro and/or nano roughness to strengthen the hydrophilicity of the implantable surface [9,111,135,136]—a typical example of contact angles for Ti, as-grown NTs and NTs annealed at different temperature is shown in Figure 8a [111]. Here, the fine-tuning of surface wettability can be achieved by either development of particular surface roughness or surface modification with low-energy materials. This is because one step is normally required to alter the hydrophilic surface to a hydrophobic one, i.e., by the deposition of low surface energy material (as exemplified in Figure 8b,c, whereby the deposition of polydimethylsiloxane, PDMS, using a ultraviolet (UV) light grafting technique turns hydrophilic TiO_2_ NTs into hydrophobic [136]). The latter was obtained by various surface modification techniques as discussed in recent reviews or the literature [136,137,138].

#### 2.2.4. Corrosion Resistance of Anodic TiO_2_ Nanotubes

Concerning the biocompatibility of implant biomaterials, in vivo corrosion resistance has a crucial contribution to the implant’s lifetime, and for metallic biomaterials (excluding the biodegradable ones) this is “the more corrosion resistant, the more biocompatible” [139]. This, as physiological solutions, are viewed as extremely corrosive towards metallic biomaterials and if corrosion occurs it can lead to diminishing biocompatibility, i.e., a release of metallic ions or particles as a result of corrosion can lead to inflammation and, finally, to tissue loss. Though usually, compared to compact biomaterials, porous materials are more prone to corrosion [140], in the case of nanostructures, both the morphology and the metal oxide itself (pure Ti or alloys) contribute to the corrosion performance [93].

**Figure 9:**
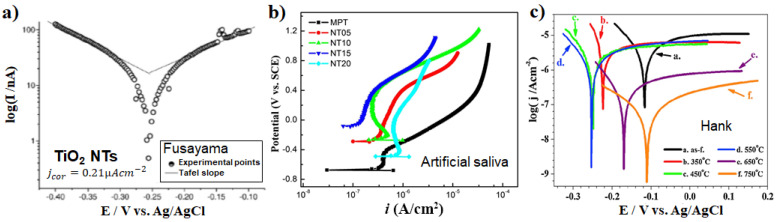
Typical Tafel plot for nanostructures on Ti measured in (**a**) Fusayama solution for TiO_2_ nanotubes (reprinted with permission from ref. [141]. Copyright 2010 Elsevier), (**b**) in artificial saliva for mechanically polished Ti (MPT) and TiO_2_ nanotubes obtained with different morphology as a result of anodization at different voltages (5, 10, 15, and 20 V) (reprinted with permission from ref. [142]. Copyright 2011 Elsevier, (**c**) in Hank’s solution for as-formed TiO_2_ nanotubes and annealed in air at temperatures of 350, 450, 550, 650, and 750 °C (reprinted with permission from ref. [111]. Copyright 2016 Elsevier).

Investigations into the corrosion performance of amorphous/annealed NT structures, either grown on Ti [111,141,142,143,144,145,146,147,148,149] or its alloys (Ti6Al7Nb [76,150,151], TiZr [152]) indicated that for all NT layers the electrochemical parameters show very stable characteristics—as shown in Figure 9 for various NT structures. E.g., in the well-established Fusayama artificial saliva solution (consider also that the NTs had a corrosion current density, $j_{cor}$ of 0.12 µA cm^−2^, while bare Ti had 7.12 µA cm^−2^) [141], another artificial saliva (showing the influence of tube morphology) [142], and Hank’s solution (evaluating the influence of the annealing treatment) [111]. For annealed NTs, lower corrosion current density (at least one order of magnitude) was recorded compared to the amorphous NTs [111,147], and this could also be linked to the appearance of rutile at the NT/metal interface). For instance, in Hank’s solution Ti is reported with a corrosion current density of 1.29 µA cm^−2^ [153] to 2.3 µA cm^−2^ [154] (and 0.116 µA cm^−2^ for smooth Ti [144]), while for the annealed NTs this is in the range of 2.51 to 0.07 µA cm^−2^ decreasing with annealing temperature up to 750 °C (see also the Tafel plots in Figure 9c) [111], or 0.014 µA cm^−2^ for anatase NTs [144]. Overall, a similar trend is maintained for the corrosion current values in the literature for the various saliva or solutions used, namely with annealing the corrosion protection of the nanotube layers increases. To note that (i) linear polarization measurements/Tafel plots are usually measured when steady-state conditions are achieved at the interface between metal/solution (e.g., the change of the corrosion potential is less than 5 mV in 10 min) [155], and (ii) at least one of the branches has to exhibit linearity on a semi-logarithmic scale over at least one decade of current density to allow the extrapolation of the Tafel slope [156,157,158] (see also the Tafel plots in Figure 9c showing linearity only in the cathodic region).

For NT structures, the tube diameter and the thickness of the barrier layer are also crucial, significantly affecting their electrochemical corrosion performance [142,145,148,159]. The linear polarization curves in Figure 9b demonstrate that the passivation currents of the different morphology NTs, i.e., increasing diameter and tube length with increasing the applied voltage, were mostly lower than for the bare Ti. It is worth mentioning that the morphology type, pore diameter, and thickness of the barrier layer are controlled by the anodization conditions [34]. Concerning morphology, NTs grown on NiTi alloys lead to lower corrosion resistance and more Ni release compared to the bare NiTi alloys, due to the increased surface area [95]. Whereas a 5.3 µm thick nanoporous layers (93 nm pore diameter) led to an improvement of the corrosion resistance [160]. In general, for NTs grown on alloys, the alloying elements and their amounts contribute substantially to the electrochemical stability of NTs [89,152]. For example, as in the case of Ti30NbxZr or Ti30TaxZr, where the corrosion resistance was improved with the increase in the Zr content from 3 to 15 wt.% [89].

Similar performance, i.e., resulting in an improvement of the corrosion resistance in artificial saliva by the formation of NT structures (amorphous), on either ultrafine-grained or coarse-grained Ti13Nb13Zr alloys was observed [161]. Saji et al. [91] reported that in the case of nanostructures on Ti35Nb5Ta7Zr alloy, namely nanopores or nanotubes with diameters of 30–50 nm or 30–80 nm, respectively, and annealed to anatase in argon atmosphere (550 °C), nanopores showed a corrosion current density of 0.76 µA cm^−2^, lower than the 3.12 µA cm^−2^ obtained for NTs (in aerated Ringer’s solution). This difference is due to the distinctly separated barrier oxide/tube bottom interface. Furthermore, also for a nanochannel morphology, a decrease in the corrosion current density was observed compared to compact oxide (e.g., for nanochannels on TiZr alloy that show partially crystalline structure due to the anodization in hot-glycerol phosphate electrolytes) [16].

## 3. Tailoring Osteoinduction with Anodic TiO_2_ Nanotubes

### 3.1. Advantages of Anodic TiO_2_ Nanotubes for Osteoinduction

#### 3.1.1. Nanotopographical Cues of Anodic TiO_2_ Nanotubes

Most known mammalian cells exhibit the instinct to adhere onto a surface to carry out normal metabolism, proliferation, and differentiation [10,162,163]. Cells anchor onto a surface and sense the extra-environment through ion channels and receptors presenting at their membranes, then integrate the chemical and physical signals from the extra-environment and give the response that some transmembrane receptors form clusters known as integrins, and associate intracellularly with groups of proteins which link them to the cytoskeleton. Subsequently, focal adhesions take place through the binding between the cluster integrin receptor and the ligand of the extracellular matrix (ECM) [133,163,164]. The cells will undergo apoptosis if are not able to synthesize and deposit their own ECM molecules in a short time [164,165].

Nowadays, much more attention has been paid to nanoscale microenvironments surrounding cells, due to the highly sensitive cell responses including cell adhesions and cell decisions for cell growth and differentiation to nanotophographic cues. Various techniques on different biomaterial systems including TiO_2_ nanostructures have been introduced to achieve surface structures in the sub-100 nm region for the investigation of cell-stimulating effects and biomimetic activation [166]. For example, geometrically defined, adhesive and stable surface protrusions were made based on polymer demixing [167], ordered gold cluster arrays [168,169], nanophase ceramics, biomedical alloys [42,170], or self-organized nanoporous aluminum surfaces. Among the biocompatible implant materials suitable for bone repair, titanium has been widely accepted as the most favorable for osteogenic differentiation in vitro and bone regeneration in vivo. One of the most clinically relevant advantages of anodic nanostructures for osteoinduction and osteogenesis is well-defined, self-organized, nanoscale topography coatings on TiO_2_ implant materials, which support the growth and osteogenic differentiation of mesenchymal stem cells (MSCs) in vitro and in vivo bone formation [7,171]. Moreover, TiO_2_ substrates with well-defined nanoscale topography allowed highly reproducible cell behavior responding to the topographic cues from the oxide surface.

Fundamental findings reported more than a decade ago, demonstrated that the diameter of anodic TiO_2_ NTs vitally influences the cell (mesenchymal stem cells—MSCs, osteoblasts, osteoclasts, etc. [34]) activities leading to increased cell adhesion and growth as compared to compact oxide or bare Ti [7,43,172]. These findings led to further research focused on diameter-controlled nanostructures for implants/tissue engineering and drug delivery design.

For the influence of NT diameter on the different cell type adhesion, proliferation, and differentiation, please see (i) the works of Brammer et al. [173] and Chamberlain et al. [21] and (ii) the works of Park et al. [7,8,35,43] and Khaw et al. [174]. Brammer et al. and Chamberlain et al. showed that small diameter NTs (30 nm) promoted the highest degree of osteoblast activity and diameters of 70 nm results in the diminished inflammatory response, respectively [21,173]. Park et al. [7,8,35,43] proposed that 15 nm NTs in diameter is an optimal tube diameter for increased cell adhesion and proliferation for MSCs and endothelial cells. Further, Bauer et al. [175] confirmed the same trend for ZrO_2_ NTs, indicating that the NT size effect dominates over the crystal and fluoride content [8]. In addition, Khaw et al. [174] showed that there is a discrepancy concerning the affinity of hMSCs and human osteoblasts (HOB) for TiO_2_ NT of various pore diameters in the sense that hMSCs prefer NT with a 20 nm diameter in terms of osteogenic differentiation while 50 nm nanotubular surfaces potentiate osteoblastic maturation of HOBs.

At the cell-substrate contact area, nanotopography mainly affects the cell responses and following behaviors including cell decisions, for example, whether cell divides or starts to be differentiated. Particularly in the nanoscale cell-substrate contact area, the size of the lateral spacing can determine cell fate depending on whether the lateral gap size allows the integrin clustering on the NTs or not. Integrins have been known as one of the most important microenvironmental signal receptors in the mammalian cell system, and integrin clustering can be developed when integrins are activated by environmental signals out of extracellular matrix/substrates. The study of Park et al. [7] showed that the gap size of around 10–20 nm in NT diameter perfectly fits the integrin clusters leading to focal adhesion complexes and downstream signaling to the nucleus. Likewise, recent studies [176,177] have brought to focus the contribution to the decipherment of the molecular mechanisms of how integrin-mediated cell interaction with TiO_2_ NTs may direct cell fate, i.e., cell adhesion and proliferation, cytoskeleton reorganization, motility, cell shape, and osteogenic differentiation.

These results led to the establishment of the osteogenic differentiation mechanism. First, smaller tube diameters of 10–20 nm provide the optimal length scale for integrin clustering and the formation of focal contacts on the nanotube surface which further result in higher cell proliferation, migration, and differentiation to osteogenic lineages of the MSCs compared to bare Ti implants or compact TiO_2_ layers. Considering the estimated occupancy size of the head of the integrin heterodimer, the 15–20 diameter of the nanotubes result in the clustering of integrins in the closest possible packing which leads to an optimal integrin activation [7,43]. In contrast, larger tube diameters (e.g., >50 nm) impaired cell spreading, adhesion and even prevented integrin clustering and focal adhesion complex formation—this led to a strikingly reduced cell behavior (proliferation, migration, differentiation) and in the end induced the adhesion-dependent form of apoptosis [7]. Secondly, on the small diameter nanotubes, phosphorylation of the focal adhesion kinase (FAK) and extracellular regulated kinase (ERK), target of the FAK signaling pathway was highest compared to on the much larger 100 nm diameter nanotubes [7].

Reports in literature confirm that different nanotopography cues (micro-scale surface modifications, nanopits, nanosheets, nanotubes, etc.) influences osteogenic differentiation via various downstream molecular pathways following integrin signaling, reorganization of the actin cytoskeleton, and nuclear translocation/transcription [10,178,179]. These include several canonical pathways such as FAK [7], ERK [7,179], phosphoinositide 3-kinase/protein kinase B (PI3K/AKT) [179], Rho-associated kinase (Rho-ROCK) [179,180], autophagy-mediated signaling between Yes-associated protein (YAP) and β-catenin [181], mammalian target of rapamycin complex with Rictor (mTORC2) [182], and Wnt/β-catenin [183] signaling pathways. Recently, Lv et al. [184] revealed the epigenetic mechanism of nanotube-guided osteogenic differentiation of MSCs and that changes in cell adhesion and cytoskeleton reorganization are linked with epigenetic alterations. The mechanism would be even more complex as the above-mentioned pathways can crosstalk and have a crucial role in the cell adhesion, migration, proliferation, and differentiation directed by the biomaterial surface.

Not only topography itself but also a minute mechanical strain on nanotube layers seems to affect the stem cells, resulting in their osteogenic differentiation [176,180,185,186,187]. This mechanical strain has been reported to promote osteogenic differentiation of MSCs on TiO_2_ nanotubes via the FAK [185], ERK1/ERK2 [186], Rho-ROCK [180], Yes-associated protein/Tafazzin (YAP/TAZ) [187] FAK-ERK1/ERK2-RunX2 [176] pathways. The exact mechanism through which nanotopography modulates mechanotransduction for osteogenic differentiation of MSCs still remains to be further confirmed through additional investigations.

Recently, we compared the generally-grown (close-packed) NTs with spaced NTs, where both NTs have similar diameters of ≈ 80 nm, but spaced NTs have a 80 nm tube-to-tube individual spacing. The spaced NT morphology did not show any detrimental effect on osteoblast functions in vitro, rather having a beneficial influence on the osteogenic differentiation of pre-osteoblasts [188], indicating that gap distances irrespective of inner or outer nanotube rims may affect cells that come into contact with these nanoscale gaps, further delivering contact signals to the nucleus to decide cell differentiation.

Many previous works demonstrated that various diameter TiO_2_ NT structures can also induce the anti-inflammatory response of hosts (Chamberlain et al. [21], Smith et al. [189], Neacsu et al. [20], Yao et al. [190], Bai et al. [191]). The surface modification induced by the nanotube structures combined with annealing can change the hemocompatibility of TiO_2_ NTs, by alleviating platelet activation (Mazare et al. [111,150], Junkar et al. [15], Huang et al. [192], Gong et al. [193], Bai et al. [191,194]). Several in vivo studies have shown that TiO_2_ nanotubular layers could induce successful peri-implant bone formation/osseointegration in various animal models using different diameter NTs: von Wilmowsky et al. (30 nm diameter nanotubes) [195], Wang et al. (30, 70, and 100 nm diameter NTs) in minipigs [174], Alves-Rezende et al. (≈ 74 nm diameter NTs) [196], Baker et al. (TiO_2_ NTs—in vitro and in vivo intramedullary fixation) in rats [197], or the review of Wang et al. [198] for the effect of TiO_2_ NTs grown on the implants’ surface on osseointegration in animal models.

Another advantage of anodic nanostructures for osteoinduction lies in the potential of a synergistic combination of nanotopographic cues with drug delivery via NT surface or inner space decoration, as a cargo. By modifying the NTs with active molecules or growth factors (such as bone morphogenetic protein-2—BMP2, epidermal growth factor—EGF, osteogenic growth peptide—OGP), improved cell adhesion or differentiation can be obtained. For example, using a BMP2 decoration, Balasundaram et al. [199] showed an increased osteoblast adhesion on TiO_2_ NTs, and Park et al. [35] reported accelerated differentiation of MSCs towards osteogenic or chondrogenic lineage on BMP2-coated NT layers. Lai et al. [200] further confirmed MSCs differentiation to osteoblasts on BMP2-decorated TiO_2_ NTs (via polydopamine). Moreover, several growth factors/cytokines can be coupled with different functional layers on TiO_2_ NTs surface [201]. For example, BMP6-loaded TiO_2_ NTs can be coated with platelet-derived growth factor containing silk fibroin in a composite for increased osseointegration [202]. Several other modifications include EGF on NT structures for increased adhesion of MSCs, OGP functionalization of the NTs for an improved cell spreading and differentiation of osteoblasts [203], and calcitonin gene-related peptide (CGRP) on TiO_2_ NTs for osteoporotic bone implants [125]. While the previous studies mostly focused on decorating growth factors or drugs on the top of NT surfaces, recent studies tend to expand drug delivery applications through the inner nanotube wall/space, as well as decorating the top surface of NTs. We will discuss this recent trend in NT drug delivery more in detail in one of the next sections.

#### 3.1.2. Electric Field Stimulation of Anodic TiO_2_ Nanotubes

In bone tissue engineering, a classic biological triad represents three critical components leading to successful bone regeneration using biomaterial implantation: (1) biomaterial/scaffolds-derived micro-, nanoenvironmental cues, (2) electrochemical, electrical, physical regulatory signals including bioreactive molecules, and (3) cells including cell-cell contact and functional matrix signaling. Among these three major components, we already discussed in a former section the TiO_2_ nanotube-derived nanoenvironmental cues affecting cell behaviors. We also shortly mentioned bioreactive molecules on TiO_2_ nanotubes, that stimulate osteogenic differentiation and osteoinduction. Considering the scope and limitation of the present review, discussion about cell components (related to point 3) including the interplay of different bone and precursor cells interacting via cell-cell contacts, is beyond the topic of the review. In this section, electrical stimulation among regulatory signals in the triad, especially on TiO_2_ nanotubes for osteoinduction will be introduced and reviewed.

In the field of bone tissue engineering using biocompatible implants, cellular behavior and cell fate of stem cells have been known to be determined not only by microenvironmental signals, such as substrate topography, soluble growth factors and cytokines, and cell–cell and cell–extracellular matrix interactions, but also by electrochemical signals [164]. Electric fields have been applied in tissue engineering for many different purposes. The main applications of electric fields are in the characterization of artificial tissues and their component cells, and the formation of artificial tissue-like materials, either by assisting in the formation of the artificial extracellular matrix (e.g., the formation of scaffolds by electrospinning), or the micromanipulation of the cells themselves using electric fields [204]. Of further potential interest in tissue engineering are also the biological effects of the electric fields. In fact, the clinical application of an electric field (EF)/an electromagnetic field for bone regeneration has a long history. A meta-analysis of the data in the last 60 years shows clinical EF trials to have an overall favorable influence on bone healing [205,206]. However, such clinical trials have also considerable variations depending on the treatment regimen and study design, thus hampering a direct comparison and a critical evaluation of results [205]. Further, despite a long history of clinical trials, the mechanism linking electric stimuli and its sensing on the cell surface is still elusive. Therefore, during recent decades, the mechanism of EF (e.g., how cells sense and react to EFs) affecting cell adhesion, migration, proliferation, and differentiation has been extensively investigated in various primary cell/cell lines [206,207,208,209,210,211] including epithelial, mesenchymal, and neural cells [212,213,214,215]. Exposure of the cells to an electric current, if a certain current value threshold is exceeded, leads to cell death, while currents lower than 11 As/m^2^ have shown no decrease in cell viability [216]. Asides from the overwhelming impact of the electric current value, there are additional aspects that can negatively influence the viability of the cells, such as local acidic pH [217], metal ions release from the electrodes or cell contact directly on the electrodes [216]. Commonly, in a physiologically tolerable EF strength (<5 V cm^−1^), many of the different cell types can immediately react to EF stimuli, which include EF directional axis-dependent cell migration (galvanotaxis), neuronal activation and regular muscle cell alignments. These EF-corresponding specific cell responses have been suggested for use in wound healing, neurons stimulation, and tissue engineering on various scaffolds [206,218].

In the bone tissue engineering field, evidence has accumulated that clearly points out that electric (direct or alternating current) or electromagnetic fields can induce osteogenic differentiation of mesenchymal stem cells or osteoblasts [210,213,215]. Recent studies have shown that various scaffolds can be used in concert with electric or electromagnetic fields for bone cell stimulation, such as (i) collagen scaffolds (with piezoelectric properties) for human osteoblasts under magnetic and additional alternating electric field [219], (ii) non-piezoelectric three-dimensional matrix for osteoblasts under electromagnetic stimulation [220], (iii) polycaprolactone based scaffolds under alternating current electric fields for osteoblast-like MG63 cells [221], or (iv) conductive polypyrrole/polycaprolactone scaffolds under electrical stimulation [222].

**Figure 10:**
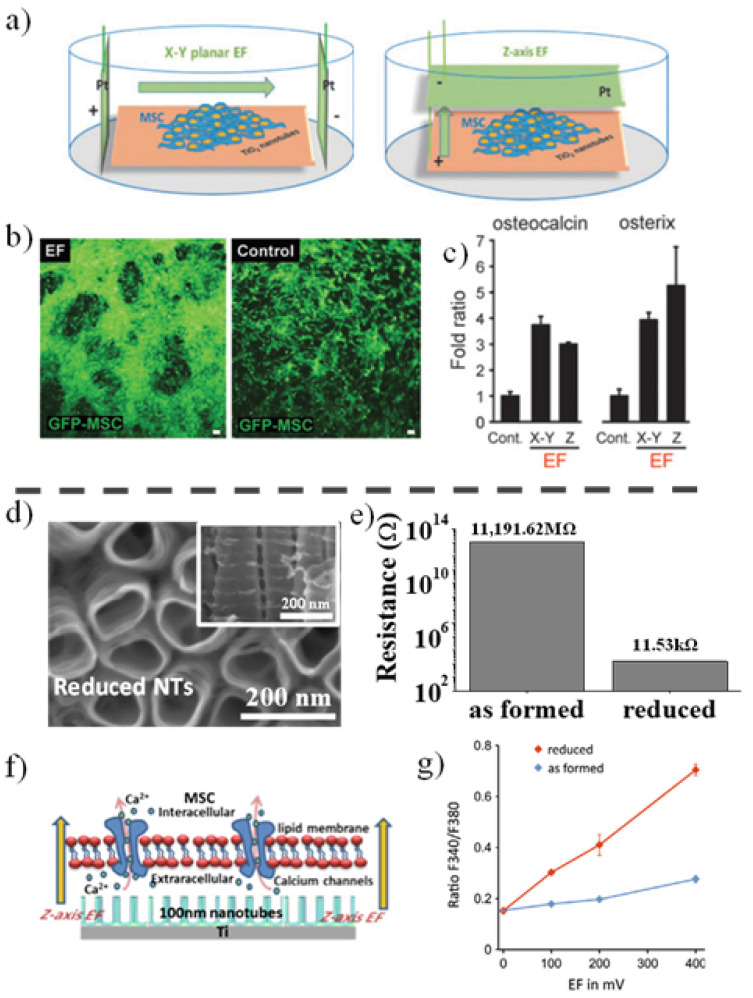
Effect of electric fields (EFs) on mesenchymal stem cells (MSCs) cultured on TiO_2_ nanotubes: (**a**) illustration of MSC under x-y planar (left), z-axis (right)-directed EF device, respectively (reprinted with permission from ref. [223]. Copyright 2016 Mary Ann Liebert, Inc.). (**b**) Osteogenic differentiation of green fluorescent protein (GFP) labeled MSCs under z-axis-directed EF (200 mV cm^−1^) for 8 days. Bar: 200 mm (reprinted with permission from ref. [223]. Copyright 2016 Mary Ann Liebert, Inc.). (**c**) Gene expressions of osteocalcin and osterix following 3 days of x-y or z-axis directed EF application by quantitative Polymerase Chain Reaction, qPCR (N = 3) (reprinted with permission from ref. [223]. Copyright 2016 Mary Ann Liebert, Inc.). Reduced NTs show higher efficiency for EF stimulation of MSCs: (**d**) SEM images of reduced 100 nm diameter nanotubes, inset: high magnification image of the tube wall structure) (reprinted with permission from ref. [224]. Copyright 2019 Elsevier), (**e**) resistance values for as formed and reduced nanotubes obtained from solid-state conductivity measurements (data taken from ref. [224]), (**f**) schematic showing the EF-triggered calcium influx (reprinted with permission from ref. [224]. Copyright 2019 Elsevier), (**g**) the voltage-dependence of the intracellular calcium elevation in as formed and reduced TiO_2_ at the end of a 10 min-EF stimulation, data were obtained from 100 randomly chosen cells/experiment and three independent experiments were performed for each group (reprinted with permission from ref. [224]. Copyright 2019 Elsevier).

Similarly, TiO_2_ substrates can also be used to stimulate bone cell differentiation under an applied EF. Already in 2016, we have reported an efficient EF-induced osteogenic differentiation of MSCs grown on 15 nm diameter TiO_2_ NT layers, without any osteogenic chemical supplements stimulating the differentiation [223]. MSCs discern the EFs and respond to both x-y planar and z-axis EFs (Figure 10a) at smaller EF strength than 0.4 V cm^−1^, i.e., at a comparable current level of endogenous provoking current in the body. Interestingly, under EF stimulation MSCs grow fast as multilayer on TiO_2_ nanotube layers while without EF they remained in a monolayer (as shown in Figure 10b).

The study further revealed that enhanced intracellular calcium signaling and the spreading of the intracellular Ca^2+^ to the adjacent cells under EF are the main mechanisms of EF-induced osteogenic differentiation. It is worth mentioning that in bone differentiation a z-axis EF using an MSC-plated TiO_2_ substrate as an anodic electrode can induce a comparable stimulating effect with x-y planar EF when MSCs were separated from EF electrodes as shown in Figure 10c (a comparison of bone differentiation-specific marker expressions). The finding indicates that MSCs can be plated or moved to adhere directly on the EF-generating TiO_2_ electrode, which might be useful for EF-engaged bone tissue engineering.

Other works also supported the stimulation of bone cells on TiO_2_ or TiO_2_ composite layers by EF, showing that electric field stimulation of osteoblast on anodic nanotubes leads to an increase in their specific biomarkers [225], and more recently Sahm et al. [226] also reported the influence of alternating electric fields on human osteoblasts growing on and in the surrounding of Ti6Al4V electrodes.

Further, a shortened EF-engaging time and an effective but minimal EF strength are critical issues in EF-applied clinical bone tissue engineering. We have reported significantly shortened EF exposure time by improving the conductivity of the TiO_2_ NTs [224]. “Black” TiO_2_ NTs, subjected to an optimal reduction treatment in argon hydrogen (Ar/H_2_) environment can lead to a significant increase in tube conductivity and decrease of the electron transport time, as a result of forming Ti^3+^ and oxygen vacancies in the NTs (see the morphology of reduced tubes in Figure 10d and conductivity values in Figure 10e). Hence, much shorter EF-exposure time (from previous days to 10 min) and a decrease in the applied EF strength (from 400 mV cm^−1^ to 100 mV cm^−1^) sufficiently enabled the EF provocation of an early response of the stem cells (a schematic drawing of increasing intracellular calcium influx under Z-axis EF in Figure 10f and a superior intracellular calcium activation on reduced TiO_2_ NTs compared to as formed NTs in Figure 10g).

Overall, in the past decade, great achievements were accomplished through many valuable studies that verified the EF mechanism (how cells recognize the EF stimuli and which signaling pathway dictates EF-induced bone differentiation). The new understanding of the mechanism may allow the accomplishment of potential clinical EF application in bone tissue engineering in the near future, following the careful optimization of EF parameters. Electric/electromagnetic field application for bone regeneration using bio-implantable materials could be a useful option when combined with the other novel technologies including TiO_2_ surface modifications and drug delivery systems which will be discussed in the following section.

### 3.2. Drug Delivery Applications Based on Anodic TiO_2_ Nanotubes

**Figure 11:**
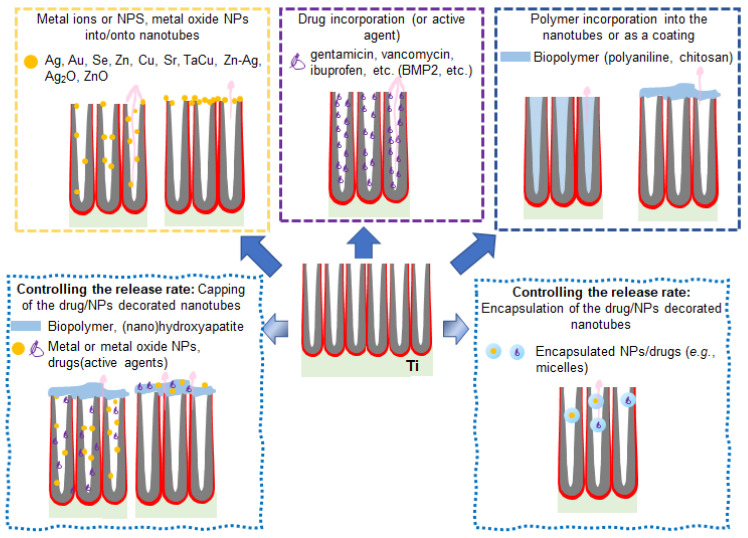
Targeted drug delivery approaches using TiO_2_ nanotubes for osteoinduction in vitro or in vivo: metal ions or NPs, drugs/active compounds, polymer coatings, and methods to control the release rate by capping with biopolymers or encapsulating the drug/active agent.

We have previously discussed the advantages of anodic TiO_2_ nanotubes on osteoinduction and osteogenesis and their validity for a wide range of Ti alloy substrates. The nanotubes’ high surface area and their distinct topography can provide their full advantage in drug/active agents delivery applications [14,227,228]. Extensive studies are investigating the release profiles of drugs, including antibiotics, peptides, metal ions (Ag, zinc - Zn, copper - Cu), or various biopolymer coatings. Overall, the studies investigating the mechanism and release rate from anodic TiO_2_ nanotube layers confirm the higher loading capabilities of the NTs (that are, as expected, linked with their high aspect ratio). Previous studies have also revealed that vacuum impregnation techniques usually lead to a higher elution time, compared to soaking techniques [229]. A variety of drug/active compound release approaches based on anodic TiO_2_ NT structures are summarized in Figure 11.

#### 3.2.1. Release Rate from Anodic TiO_2_ Nanotubes

In the first step, we will discuss key aspects concerning the release rate of drug/active components. Generally, the drug release rate is determined by the design of the delivering platform (varied from the incorporation of metal ions/nanoparticles on the surface to a direct incorporation into the TiO_2_ NT space) and biochemical characteristics of drugs: see, for example, Ag [230,231], strontium - Sr^2+^ [149], magnesium (Mg) and silicon (Si) [93], vancomycin [230], ibuprofen [127] gentamicin [127,232], doxorubicin [229,233], anti-inflammatory drugs (indomethacin [234], sodium naproxen [235]) co-delivery of drugs (gentamicin and ibuprofen) [236], or various active molecules (e.g., quercetin [237]). Usually, one of the main challenges in the design of drug delivery systems based on either TiO_2_ NTs or other nanostructured materials is a controlled and sustained release of drug in contrast to a burst release that can rapidly lead to the accumulation of the active drug/active agent to the toxic levels at the targeted site [18]. Table 1 represents an overview of drug/active agents with specific loading and release strategies using anodic nanostructures.

Jarosz et al. [127] clarified the drug release kinetics and mechanism using a desorption-desorption-diffusion (DDD) model of the drug release. In their study for ibuprofen/gentamicin release from TiO_2_ NTs, two different drug release kinetics were shown: drug desorption from the top of the nanostructure, and then desorption and diffusion of the drug from the inner nanostructure. A first-order kinetics is responsible for the initial fast drug delivery (from the surface of the nanostructure), while the slow release of the drug from within the nanostructure combined the first-order kinetics with a Higuchi model [236]. Further, the work of Pawlik et al. [236] has shown that release rate can be differentially controlled according to the characteristics of drug combinations. The co-delivery of ibuprofen and gentamicin, moderate water-insoluble and water-soluble, respectively, enables controlling the release time from TiO_2_ NTs when the loading procedure consisted of first gentamicin and then ibuprofen serially, leading to a much slower release of gentamicin thus avoiding an initial overdose burst.

Coating methods can also contribute to prolong the drug release rate, for example (i) capping techniques, such as doxorubicin loaded in NTs with a polyethylene glycol layer coating as a barrier [233], chitosan coatings of NTs loaded with indomethacin [234], gentamicin [238], selenium [239], quercetin [237] or mixed coatings of gelatin/chitosan [240], (ii) deposition of the ions on top of polymer layers (for example, Ag on polydopamine decorated TiO_2_ NTs [231]), or (iii) for encapsulation of the drugs in micelles and their loading into the NT structures (indomethacin [234]).

#### 3.2.2. Drug Delivery for Antibacterial and Osteoinductive Activities

For drug delivery on TiO_2_ nanotubular bio-implants, so far a high number of investigations has mainly focused on antibacterial effect using (i) metal ions or nanoparticles (Ag [241,242,243], silver oxide - Ag_2_O [244,245], gold - Au [122], selenium - Se [239], Zn [246], zinc oxide - ZnO [247], Cu [248], Sr [249,250], tantalum (Ta) and Cu [124], Zn-Ag [251], Sr-Ag [250], or calcium and phosphorus - Ca-P [252,253]), (ii) drugs (vancomycin [36], gentamicin [254], metformin [255], alendronate [256], simvastatin [257]) or (iii) polymers (polyaniline [258], chitosan [210,238]) to provide a contamination/infection-reduced condition beneficial for osseointegration (see Figure 11 and Table 2).

Briefly, for Ag nanoparticle (NP) decoration, deposition is achieved via: (i) a combination of solution deposition and subsequent photodeposition or UV-induced photoreduction [242,250], which can also be available for depositing the Ag^+^ ions on polydopamine decorated TiO_2_ NTs [231], or (ii) solution deposition and reduction by gluconolactone (reducing the silver ammonia to Ag NPs) [243], and more recent approaches such as (iii) anodizing alloys (TiNbAg [242] and TiNbAgPt alloys [93]), or (iv) ion implantation (graded Ag incorporation into TiO_2_ NTs by silver plasma immersion ion implantation [126] or the mixed Zn-Ag ion co-implantation [251]). While most of the works focus on the antibacterial activity of Ag decorated NTs (see also Coman et al. [259]), some researchers investigated also the interactions between Ag and host cells in view of biocompatibility. While an Ag overdose strongly influences biocompatibility due to increased toxicity against the host cells and surrounding tissue [126,241], if the Ag amount is optimal, the Ag-decorated NTs have been reported to show a similar cellular response as the undecorated NTs with epithelial cells and fibroblasts in vitro and a minimal inflammatory response in vivo [126]. Moreover, Taipinia et al. [242] reported that anodic NTs grown on TiNb alloys containing Ag have antibacterial activity without a detrimental effect on MC3T3-E1 pre-osteoblasts rather promoting cell proliferation.

Gao et al. [244] investigated Ag_2_O NPs-embedded NT structures (size 5 to 20 nm, and 0–15 at.% Ag) by a combination of sputtering titanium and silver on Ti and subsequent anodization, showing adequate antibacterial properties without cytotoxicity. The crystallized Ag_2_O NPs were embedded in an amorphous TiO_2_ NT wall enabling the sustained and slow Ag^+^ release, thus minimizing the cytotoxicity and ensuring a long-lasting antibacterial activity. More importantly, they showed no appreciable influence on the osteoblast viability, proliferation, and differentiation compared to the Ag-free bare nanotubes. Similar results of a bactericidal effect against *Escherichia coli* without detrimental effects on human osteoblast proliferation were reported when the Ag_2_O NPs were deposited by physical vapor deposition on NTs grown on Ti6Al4V [245].

Strontium represents another widely used element for functionalization of biomedical materials due to its dual mode of action on bone cells, namely, stimulation of osteoblast proliferation and differentiation, and inhibition of osteoclast function [260,261]. Sr^2+^ decoration of the NTs via a hydrothermal treatment has been reported to increase the osteobonding ability of the materials based on in vitro experiments with Saos-2 osteosarcoma cells and MC3T3-E1 pre-osteoblasts, respectively [249,250]. It is worth noting that in the latter study, Pan et al. [250] additionally incorporated Ag NPs onto Sr-loaded NTs by adsorption of Ag^+^ from 0.02 AgNO_3_ solution and subsequent UV irradiation. The simultaneous presence of Sr and Ag endowed the materials with excellent antibacterial properties and osteogenic capability in terms of pre-osteoblast adhesion, proliferation, and mineralization, as well as gene expressions of osteoblast-specific markers.

A similar hydrothermal treatment in Zn acetate solution results in Zn decoration of the TiO_2_ NTs with very good antibacterial activity and biocompatibility—while an optimum sample in respect of tube diameter (80 nm) and the time of the hydrothermal treatment (3 h) with optimal Zn release significantly enhances the osteogenic differentiation of the MSCs due to increased matrix protein deposition [246]. In addition, Jin et al. [251] reported that a simultaneous Zn and Ag co-decoration of the NTs (by plasma immersion ion implantation) results in Ag NP decoration on a Zn layer covering the NTs and this co-decoration increases initial adhesion and spreading, proliferation, differentiation, and osteogenesis of MSCs coupled with a long-term antibacterial effect (compared to Ti, or Ag or Zn single deposition), in both in vitro and in vivo experiments (due to formation of Ag-Zn micro-galvanic couples [262]). ZnO NPs decorated on NTs have also an antibacterial effect, and once the optimal loading is exceeded its efficacy can be tackled with further co-doping of Ag [247]. In a most recent study, Chen et al. [263] reported on the fabrication of Zn-incorporated TiO_2_ surfaces and their influence on the osteogenic microenvironment and bone formation. In their culture model, a MC3T3-E1 pre-osteoblasts cell line was grown in the conditioned media (CM) derived from a RAW 264.7 macrophages cell lines cultured in standard or pro-inflammatory (stimulation with lipopolysaccharide—LPS) conditions on these Zn-incorporated TiO_2_ surfaces. The results showed that macrophages cultured on Zn-incorporated TiO_2_ NTs display a M2 phenotype, while M1 markers were moderately inhibited, as compared to the LPS group. The pre-osteoblasts grown on Zn-incorporated NTs incubated in CM showed increased cell adhesion and proliferation, as well as osteogenic differentiation in comparison to their TiO_2_ NTs counterparts and the Ti group. The authors hypothesized that superimposing Zn onto a titania NT surface could increase the osteogenic potential of osteoblasts through the improved immunomodulatory function of macrophages. The study thus brought to attention the crucial roles of Zn in both bone homeostasis and regeneration [264] and in innate and adaptive immune systems [265].

Cu is a well-known antibacterial agent that is widely used in biomedical applications with other biomaterials and has anti-inflammatory, anti-microbial, and anti-proliferative properties [266]. More recently, the focus was on developing TiCu alloys, and their in vitro and in vivo evaluation corroborated their good biocompatibility and osteogenesis ability (e.g., 5 wt.% Cu content [267,268], or 10 wt.% Cu [248]). TiCu alloys enhanced the expressions of osteogenesis-related genes (including alkaline phosphatase - ALP, Collagen I, osteopontin - OPN, and osteocalcin - OCN) in vitro [268] and promoted the surrounding bone-bonding (bone-to-implant contact) and the osteogenesis in vivo [267]. In addition, Wang et al. [248] has reported the anodization of TiCu alloys (containing 90-x % Ti, 10% Cu and x Al, with x = 0.45) to have excellent antibacterial activity and minimal cytotoxicity on osteoblast cells. Currently, Wu et al. [124] has shown that a multifunctional TaCu coating on anodic TiO_2_ by magnetron sputtering (Ta:Cu 1:1 at.% ratio) enables effective bacteriostatic properties with distinct angiogenesis compared to bare NTs or a Ta coating only.

During the electrochemical anodization, ions such as Ca and P can be incorporated into the anodic nanostructures by anodizing in an electrolyte containing simulated body fluid (SBF) and subsequent cathodic deposition of Ag. This resulted in a functionalized NT layer with calcium, phosphorus and silver (Ca-P-Ag), which can enhance bone-like apatite formation in SBF and stimulate cell adhesion and proliferation of murine pre-osteoblast cells accompanying inhibition of the bacterial growth [252]. Similar results can be achieved by thin Ca-P layer deposition on NTs via immersion in Hank’s solution, followed by nano-Ag deposition by magnetron sputtering [253].

For Ag NPs decoration of NTs, a nano-hydroxyapatite co-decoration can be employed too [243], which has been reported to increase the biocompatibility and improve the control of the release rate [269,270]. A similar approach can also be used for the incorporation of drugs [36,256]. As depicted in Figure 11, polymers can be employed for filling/capping of the NTs to enhance the biocompatibility or to control the drug release (polyaniline [258], polyethylene glycol [233], chitosan [234,238] or gelatin and chitosan [240,257] as a capping agent).

For targeting antibacterial effect combined with an improved osteoinduction, the release mechanism and release rate of gentamicin [254,271], metformin [255], ibuprofen [37] from TiO_2_ NTs have been extensively studied [127,229,236]. Further, a variety of combination sets of drugs and NT decorations has been designed and suggested to get the synergistic effects on antibacterial effect and improved osteoinduction. Draghi et al. [254] confirmed that loading gentamicin into the smaller diameter (31 nm) NTs resulted in a protracted release and antibacterial action together with improved cell adhesion and proliferation of an osteosarcoma cell line. Lai et al. [257] tailored the release of simvastatin from chitosan/gelatin-coated TiO_2_ NTs improving osteoblast differentiation and inhibition of osteoclastic differentiation (compared to free-drug bare NTs).

BMP2-loaded TiO_2_ NTs with a hyaluronidase-sensitive-multilayer coating consisting of chitosan, sodium hyaluronate-lauric acid, chitosan, and gelatin have been reported to have good biocompatibility, higher cell viability, mineralization capability, and antibacterial effect [272].

Metformin-loaded NTs with a 15 layers chitosan deposition to control the drug release led to a long-term release rate of up to 21 days and significantly decreased the burst release while promoting cell attachment and proliferation of MSCs [255].

#### 3.2.3. In Vivo Drug Delivery Approaches Using Anodic TiO_2_ Nanotube Implants

In contrast to abundant in vitro research works investigating the advantages of TiO_2_ NTs as drug delivery systems, only a limited number of studies progressed to in vivo drug delivery experiments on animal models using nanostructured implants based on TiO_2_ NTs (please see Table 3).

The most recent studies have been focused on growth factors applications that can endow the NT surfaces with additional benefits in terms of implant osseointegration. BMP2, which was proven as the most potent stimulator in inducing bone regeneration [280,281], has been widely used to functionalize TiO_2_ NTs. One of the well-designed in vivo studies performed by Lee et al. [38] established anodic TiO_2_ NTs (with diameters of ~70 nm and ~110 nm, respectively) decorated with rhBMP2 by dip coating. The in vivo study was performed on the following implant groups in New Zealand white rabbits: (Group 1) a machined surface; (Group 2) a sandblasted large-grit and acid-etched SLA implant (as a positive control group); (Group 3) TiO_2_ NT; and Group 4) TiO_2_ NTs with rhBMP-2. Histomorphometric/micro-computed tomographic analysis at 8 weeks post-implantation showed that Group 4 obtained the highest BIC and bone volume ratio. As a conclusion, the authors suggested the designed NT drug delivery platform as a promising reservoir that allows a slow and sustained rhBMP2 release to reinforce implant osseointegration.

In line with this, Zhang et al. [273,274] proposed a new strategy to functionalize TiO_2_ NTs by loading a lentiviral vector encoding BMP2 (Lenti-BMP2) by lyophilization following trehalose addition. The obtained TiO_2_-Lyo-Tre-BMP2 nanoplatform ensured a slow and prolonged BMP2 release and promoted the highest extent osteogenic differentiation of bone marrow stromal cells as well as anti-inflammatory activity. The studies suggested that the delivery of BMP2 using the Lyo-Tre-based system is an effective method avoiding the adverse effects induced by the administration of high doses of BMP2.

In other studies, the osseointegration capability of BMP2 was compared with other bioactive agents such as the anti-inflammatory drug ibuprofen [37] (see also Table 2). The in vivo study [37] composed of four implant groups (conventional, NT coated, rhBMP2 loaded-, and ibuprofen loaded-TiO_2_ NTs) showed that the histological analysis at eight weeks after implantation surprisingly revealed the highest BIC ratio (71.6%) in the ibuprofen-loaded group, while rhBMP2 loaded implants showed a significantly decreased BIC (24.6%). The authors explained this effect by the osteoclastic bone-resorbing activity of rhBMP2, indicating that careful fine-tuning may be required to get the optimal dosage of rhBMP2 for successful osseointegration.

Another growth factor widely investigated for bone regeneration is platelet-derived growth factor-BB (PDGF-BB) [275], as it can stimulate the recruitment, proliferation, and osteogenic differentiation of osteoprogenitor cells or MSCs as well as the angiogenesis process [282]. Zhang et al. [275] applied rhPDGF-BB on 70 nm diameter TiO_2_ NT surfaces arrays by vacuum extraction for in vivo osseointegration in ovariectomized (OVX) rats as an osteoporosis-induced animal model. Protein particles aggregated on the surface and inside NTs could be slowly released for at least 14 days without losing their bioactivity. A higher rhPDGF-BB immobilization to the underlying TiO_2_ NTs substrate by vacuum extraction method led to an enhanced cell adhesion, proliferation, and osteogenic differentiation in vitro. Further, an in vivo study of osseointegration (oxalic acid-etched Ti group; TiO_2_ NTs modified Ti group; PDGF-coated TiO_2_ NTs group; and PDGF coated TiO_2_ NTs + Vacuum group) showed that the NTs loaded with rhPDGF-BB under vacuum improve the implant fixation ability and the rapid new bone formation in OVX rats, suggesting a novel implant coating strategy in the treatment of bone defects associated with osteoporosis.

To endow the NT surface with a local anti-osteoporosis property, Shen et al. [256] proposed a loading of alendronate (ALN), a powerful anti-osteoporosis compound that is largely used in clinics due to its antiresorptive capacity [283], onto hydroxyapatite-TiO_2_ NT substrates (NTs diameter 70 nm, length 0.7–1.0 µm). The resulted material (NTs-HA-ALN) has shown great potential in improving the proliferation and osteogenic differentiation of osteoblasts isolated from neonatal rat calvaria and inhibiting differentiation of RAW 264.7 in mature osteoclasts, compared to bare Ti, TiO_2_ NTs, and TiO_2_ NTs deposited with nano-HA layers (NTs-HA). Furthermore, in vivo tests with osteoporotic rabbits, attested the highest potential of NTs-HA-ALN implants to enhance the local osseointegration with excellent pro-osteogenic and anti-osteoporosis properties at three months after implantation, considered as the synergistic effects of the release of Ca^2+^ and ALN.

Natural compounds such as propolis [279] and icariin [276,284,285] have also been introduced as another strategy of surface functionalization to increase the in vivo performance of TiO_2_ NTs. Propolis is a natural compound collected by honeybees from various plants whose biological activity mainly depends on the flavonoids from the polyphenolic fractions, followed by aromatic acids, phenolic acid esters, etc. [286]. Its beneficial effects on bone healing are well known and attributed to its anti-inflammatory, antioxidant, and anti-osteoclastic activities [287]. Somsanith et al. [279] loaded propolis (PL) on anodic TiO_2_ NTs (PL-NTs-Ti) and showcased their ability to sustain the viability and differentiation of MC3T3-E1 pre-osteoblasts and in vivo osseointegration in a rat mandibular model. Besides enhanced cell viability and alkaline phosphatase activity in cell culture experiments, the PL-NTs-Ti resulted in enhanced formation of new bone and increased mineral density in the region surrounding the implant as well as higher expressions of collagen fibers, and BMP2/BMP7 as compared to the drug-free TiO_2_. Moreover, the peri-implant expressions of the pro-inflammatory cytokines (IL-1ß, and TNF-α) were significantly reduced, suggesting that the propolis-functionalized nanostructure also has the potential to inhibit early inflammation and block peri-implantitis.

Ma et al. [276] functionalized TiO_2_ NT surfaces with icariin (ICA, a Herba Epimedii derived flavonoid with osteogenic and anti-osteoclastogenic effects [288]) and poly lactic-co-glycolic acid (PLGA). The resulting NTs-ICA-PLGA substrate was able to ensure a sustained drug release up to two weeks with in vitro best osteogenic differentiation and in vivo higher bone formation area percentage compared to other groups during the early-stage of osseointegration. Another approach to improve the osseointegration of TiO_2_ NTs was the incorporation of Sr and icariin onto the anodized Ti surface through hydrothermal treatment and vacuum freeze drying, respectively [284]. In this study, the authors demonstrated that icariin loading onto Sr-containing TiO_2_ NT surfaces exerted additional positive effects on the pre-osteoblast behavior in terms of proliferation and osteogenic differentiation, as well as on the bone formation around screw-shaped Ti-based implants. These effects can be ascribed to the icariin ability to guide bone regeneration and promote osteogenesis [289] combined with the dual effects of Sr on bone tissue through stimulating the proliferation and differentiation of osteoblasts and inhibiting the osteoclast activity [290,291], implying that the ICA-Sr-TiO_2_ coating may be a potentially useful option in osteoporotic patients.

Si, another bioactive trace element, possesses a bone affinity that has been shown to enhance osteoblast proliferation and differentiation, stimulate collagen synthesis and bone mineralization [292,293], and to further inhibit the osteoclastogenesis and bone resorption processes [294]. Zhao et al. [277] fabricated Ti substrates (discs and screws) modified with silicon doped TiO_2_ NTs (Si-TiO_2_-NTs) by in situ anodization and Si plasma immersion ion implantation (PIII) technique and compared their activities with those for Ti and TiO_2_ NTs. The in vivo results, in line with the in vitro findings, showed that the Si-TiO_2_-NTs surface increased pre-osteoblast cell proliferation and differentiation, indicated extensive bone apposition between the screw threads at the spongy level on this surface and the formation of more new trabecular bone as compared to bare Ti and TiO_2_-NTs screws.

Considering that chronic implant-associated bone infections represent a major problem in orthopedic and trauma-related surgery due to the severe complications in the affected patients, increasing interest has been given to design surfaces with bacteriostatic and bactericidal properties. With this purpose, many antibacterial agents such as antibiotics, metal ions, anti-microbial peptides, and biopolymer coatings [14,227,295,296] were incorporated into TiO_2_ NTs and investigated for their therapeutic efficiency, mostly by in vitro antibacterial and cell culture-based studies. A very recent paper by Wu et al. [278] has revealed the antibacterial potential of polyhexamethylene guanidine (PHMG) coated TiO_2_ NTs (PHMG-NTs) in an animal model implanted with *Staphylococcus aureus*-contaminated rods in the femoral medullary cavity. This eco-friendly polymer was shown to exhibit a high and broad-spectrum antibacterial effect, which can efficiently inhibit bacterial biofilm formation [297,298] with improved bone-forming capacity.

## 4. Conclusions and Outlook

Nanoscale surface modification of Ti or Ti-based alloys via electrochemical anodization, resulting in TiO_2_ nanostructures including nanotubes (NTs), has gained significant recognition and insight in biomedical applications. Herein we discussed the current state-of-the-art in NT morphology (nanotube, nanopore, mesoporous) and their synthesis (closed-packed, open-top, and spaced NTs or long-range ordered NTs), with the critical aspects affecting the drug delivery. Further, we reviewed the properties of anodic NTs including crystallinity, surface roughness, wetting characteristics, and corrosion resistance.

Next, we evaluated the key interactions of anodic TiO_2_ nanostructures with bone-related cells, such as osteoblast cells and mesenchymal stem cells, emphasizing the influence of the nanoscale topography on the interactions with these cells. In addition, a more recent approach, highlighting the synergistic effects of electrical stimulation on cells combined with the nanoscale environmental cues from TiO_2_ NTs for enhancing bone induction was further discussed. Such a synergistic approach would be promising for tissue engineering applications using nanostructured scaffolds materials based on anodic TiO_2_ NTs with concomitant drug delivery and/or electrical/electromagnetic stimulation.

For dental or orthopedic implants, anodic TiO_2_ NTs and nanostructures with surface modifications delivering specific targeting drugs are shown to be clinically useful approaches for superior osteoinduction and successful osseointegration, especially in medically compromised patients including osteoporosis, chronic inflammatory, and metabolic diseases in the future. For successful osseointegration, nanoscale topographical cues tuning by nanoscale lateral spacing can be a powerful stimulator when combined with the specific nanotubular/nanoporous shape and high surface area as a delivery platform, allowing decoration/incorporations of antibacterial agents and/or other drugs/active agents with time-scheduled drug release rate. The release rate can be further improved avoiding an initial burst release, either by the use of polymer capping or by tuning the morphology of the nanostructures. We discussed in detail the various modifications of anodic NTs with metal ions, nanoparticles, drugs, growth peptides for controlling the drug release rate and enhancing antibacterial and osteoinductive properties, both in vitro and in vivo.

Overall, in recent decades, we fully recognized the nanoscale significance of bio-implantable substrate surface topography on a local wound healing and bone regeneration, and now more clinicians/researchers in tissue engineering fields are excited to find out which combinations of stimuli including nanoscale topography, electrical, and/or biomolecular approaches could provide a best-fit synergistic effect on bone tissue engineering.

## Figures and Tables

**Table 1:** Overview of drug/active compounds loading and releasing from anodic nanotubes on Ti or Ti alloys (D—diameter, L—length).

**Drug**/ **Compound**	**Nanostructures**		**Drug/Agent Deposition**	**Release Rate**	**Reference**
	**Type**	**D, L**			
Sr^2+^	TiO_2_ NTs (mainly anatase)	D: 110 nm L: 2.1 µm	Sr^2+^—dip coating	A viable alternative in orthopedics to provide improved corrosion resistance and enhanced biocompatibility	[149]
Ag or Vancomycin (VAN)	TiO_2_ NTs (anatase, rutile)	Aqueous: (a) D 70 nm, L 0.87 µm (b) D 100 nm, L 1.45 µm. Organic: L 6.5 µm	vacuum impregnation technique for both VAN or Ag, from 10% VAN or 10% silver nitrate (AgNO_3_) solutions, respectively	VAN release was significantly retarded from NTs in organic electrolytes (compared to aqueous). Ag release was retarded from aqueous nanotubes compared to Ti surfaces.	[230]
Gentamicin (GEN)	TiO_2_ NTs on coarse or ultrafine-grained Ti	D: not specified L: 8 µm or 15 µm	immersion of samples in phosphate-buffered saline solution containing GEN	Partly delayed release of gentamicin, for targeting bacterial inflammation around the implant.	[232]
Ibuprofen (IBU) or Gentamicin (GEN)	TiO_2_ NTs Amorphous Anatase Anatase and rutile	D: 65 nm L: 2.1 µm	10 wt.% solution of IBU in methanol, and 10 wt.% GEN in water. 5× of 1 mL pipetting and drying (drying in air, room temperature or 75 °C)	The release process is governed by the desorption of the drug from the top surface, followed by a combination of desorption and slow diffusion of the drug from the inside of the nanostructure.	[127]
Doxorubicin (DOX)	TiO_2_ NTs (amorphous)	D: 110 nm L: 0.80 µm	(a) immersion of samples in DOX solution (soaking) or (b) vacuum impregnation (drying under vacuum, several times)	DOX loaded by soaking, the elution time is around 7 days, while for wet vacuum impregnation it reached 30 days.	[229]
	TiO_2_ NTs (amorphous)	D: 170—220 nm L: 0.93 µm	15 cycles of deposition and drying under vacuum in DOX solution. Polyethylene glycol (PEG) layer for capping (1 to 50% PEG)	Release of doxorubicin can be controlled (slowed down) only during the first 3 h by the PEG layer. TiO_2_ NTs are competitive for drug release of low polarity drugs compared to other boron or carbon-based materials.	[233]
Ibuprofen (IBU) and Gentamicin (GEN)	TiO_2_ NTs (anatase)	D: 49 nm L: 0.5, 0.8 or 1.8 µm	10 wt.% solution of IBU in methanol, or GEN in water were prepared. 1 mL of each solution: a) IBU and GEN at the same time (de-noted IG), b) GEN and then IBU (GI), c) IBU and then GEN (I&G)	The length, crystallinity, and loading procedure of NTs influence the drug loading and release processes. Drug release can be modified by the loading procedure (GI approach led to the longest period or release time for GEN as the initial burst release was inhibited).	[236]

**Table 2:** Overview of drug or active compounds delivery applications using anodic nanostructures on Ti or Ti alloys (D—diameter, L—length).

**Drug**/ **Compound**	**Nanostructure**		**Drug/Compound Deposition**	**Biological Effects**	**Reference**
	**Type**	**D, L**			
Sr^2+^, Ag	TiO_2_ NTs (anatase)	D: 70 nm L: not specified	Sr^2+^—hydrothermal treatment; Ag^+^ by photodeposition to Ag NPs	Enhancement of the osteobonding capability of the nanotubes, as well as of their antibacterial activities by combining the pro-osteogenic effects of Sr^2+^ and strong antibacterial effect of Ag NPs.	[250]
Ag_2_O	TiO_2_ NTs (amorphous)	D: 80 nm L: 6 µm to 2 µm, decreasing with Ag %	Ag_2_O nanoparticles are embedded into the nanotubes. Substrates are TiAg layers (magnetron sputtering)	Sustained antibacterial activity due to the controlled low dose Ag^+^ release, improved cell attachment and spreading, no deleterious effects on pre-osteoblast cell viability, proliferation, and differentiation.	[244]
Zn	TiO_2_ NTs (30 nm: anatase, rutile; 80 nm: anatase)	D: 30 nm, 80 nm L: not specified	Zn—deposition onto the NTs by hydrothermal treatment	Antibacterial effects depending on the amount of loaded and released Zn in NTs. 80 nm NTs (3 h Zn deposition) enhance MSC osteogenic differentiation (enhanced protein deposition, enabling cell functionalities and Zn release).	[246]
Polyaniline (PANI)	TiO_2_ NTs (anatase/rutile)	D: 85 nm L: not specified	PANI deposition by electropolymerization	PANI/TiO_2_ NTs supported the viability/proliferation of MG-63 osteoblasts and showed good anti-biofilm activity.	[258]
Se-Chitosan	TiO_2_ NTs (amorphous)	D: 110 nm L: 0.90 µm	Se is deposited by electrodeposition and Chitosan by spin coating	NTs-Se-Chi samples showed excellent antibacterial activity and promoted the proliferation and biological functions of healthy osteoblasts while inhibiting the growth of cancerous osteoblasts.	[239]
Metformin (MET)-Chitosan	TiO_2_ NTs	D: 160 nm L: ≈9–10 µm	5 cycles of deposition-drying in air of MET solution in fetal bovine serum. Chitosan was deposited by spin coating	A 15-layer chitosan deposition could prolong the metformin release up to 21 days (with a significant decrease in the burst release), while the chitosan coating of the MET-loaded TiO_2_ NTs increased MSCs attachment, proliferation, and differentiation.	[255]

**Table 3:** Overview of bioactive compounds delivery platforms using anodic TiO_2_ nanostructures in the animal in vivo models (D—diameter, L—length).

Bioactive Compound	Implant Characteristics	Drug Loading Method	Animal in Vivo Model/Biological Effects	Reference
rhBMP2	TiO_2_ NTs D: ~70 nm, ~110 nm; Implant: D 3.5 mm; L 8.5 mm	Dip-coating in 1.5 mg rhBMP-2/mL (in a vacuum chamber)	Pilot in vivo study: New Zealand white rabbits, 4 types of implants (proximal tibia); rhBMP2-loaded implants: the highest BIC and enhanced bone remodeling.	[38]
rhBMP2/ Lenti-BMP2	TiO_2_ NTs: D ~100; L: 400 nm; Ti rods (D: 2 mm; L: 8 mm)	Lyophilization of Lenti-BMP2 in the presence of trehalose	Femur defect model in Fisher 344 rats; TiO_2_-Lyo-Tre-BMP2 implant: most effective in terms of BMP2 stability, sustained release, bioactivity, bone regeneration.	[273,274]
rhBMP2 and Ibuprofen (IBU)	TiO_2_ NTs: D ~70 nm; L: 5 µm; Ti rods (D: 2 mm; L: 8 mm)	IBU (1.5 mg/mL) and rhBMP-2 (10 mg/mL) loading by dip coating (3×), lyophilized, freeze and vacuum dried.	IBU-NTs behaved as an anti-inflammatory drug and improved the osseointegration of orthodontic miniscrews in vivo. However, the effect of rhBMP2-loaded NTs on the osseointegration was slightly lower.	[37]
rhPDGF-BB	Ti rods (D: 2 mm; L: 8 mm) NTs: D—70 nm	Immersion in 100 μg/mL rhPDGF-BB at RT (PDGF group) or put in the vacuum pump (PDGF + Vacuum group) for 10 min	OVX rats with bilateral femurs were used for the implantation; the newly designed coating contributed to the new bone formation surrounding the implant and enhanced bone fixation in OVX rats showing great promise for clinical applications in osteoporotic patients.	[275]
Alendronate (ALN)	Ti rods (D: 3 mm; L: 13 mm); NTs D: 70 nm; L: 0.7–1.0 µm (anodic oxidation), HA layers: alternate immersion method on TiO_2_ NTs surface.	NTs-HA-ALN implant: immersion into ALN 20 mg/mL solution at RT (12 h). NT-ALN implant: physical absorption of ALN on TiO_2_ NTs	Implants into the femoral epiphysis of OVX female New Zealand white rabbits. NTs-HA-ALN implants showed great potential for increasing osseointegration as compared to Ti, NTs, and NTs-HA implants, with the highest anti-osteoporosis potential	[256]
Icariin (ICA)	Cylindrical implants (D: 1.5 mm, L: 2 mm); Anodic TiO_2_ NT (D: 80 ± 10 nm)	Immersion in ICA solution (2 days), drying at 37 °C (1 day): PLGA coating (twice in a dropwise manner)	Sprague Dawley rats received implants in the femora’s mid-diaphysis; TiO_2_ NT structure and ICA synergistically promote osteoblasts’ function and PLGA coating endowed the implant surface with better osteogenic/osseointegration ability.	[276]
Silicon (Si)	Ti screws, inner/ outer D: 1.7/2 mm, L: 10 mm. Anodic NTs: inner/ outer D 60/80 nm.	Si plasma immersion ion implantation (PIII) method	Sprague Dawley rats received implants in the distal femur in the horizontal direction; Si-TiO_2_-NTs induced enhanced early osseointegration positive effect on implant osseointegration and trabecular microarchitecture formation.	[277]
Polyhexa-methylene guanidine (PHMG)	cp-Ti rods (D: 3.175 mm; L: 1.5 ± 0.1 cm; Anodic TiO_2_ NTs: D—46.4 ± 5.9 nm, L—650–800 nm.	Addition of 100 μL of 25% PHMG aqueous solution onto rods dropwise and drying by a vacuum oven at RT for 1 h (×10 times).	Rabbits implanted with S. aureus-contaminated rods in the femoral medullary cavity; PHMG-NTs showed an excellent capacity to prevent bacterial infections, as well as to promote osteogenic differentiation by increased expression of osteogenic-related genes in the femur tissues around the implants.	[278]
Propolis (PL)	Ti rods (D: 0.85 mm; L: 4.5 mm) screw-processed at a thread angle of 20 degrees; anodic TiO_2_ NT (D: 60–90 nm).	Immersion in propolis solution for 24 h at 25 °C followed by vacuum-drying at 25 °C for 24 h	Sprague Dawley rat mandibular model; increased new bone formation and mineral density around the PL-NT-Ti implant; enhanced osteogenic differentiation and increased expression of collagen fibers while pro-inflammatory markers decreased	[279]

## References

[1] Geetha M., Singh A.K., Asokamani R., Gogia A.K. (2009). Ti based biomaterials, the ultimate choice for orthopaedic implants—A review. Prog. Mater. Sci..

[2] Zhang L.C., Chen L.Y. (2019). A Review on Biomedical Titanium Alloys: Recent Progress and Prospect. Adv. Eng. Mater..

[3] Sidambe A.T. (2014). Biocompatibility of advanced manufactured titanium implants—A review. Materials.

[4] Boyan B.D., Lotz E.M., Schwartz Z. (2017). Roughness and hydrophilicity as osteogenic biomimetic surface properties. Tissue Eng. Part A.

[5] Wagner V., Dullaart A., Bock A.K., Zweck A. (2006). The emerging nanomedicine landscape. Nat. Biotechnol..

[6] Cavalcanti-Adam E.A., Micoulet A., Blümmel J., Auernheimer J., Kessler H., Spatz J.P. (2006). Lateral spacing of integrin ligands influences cell spreading and focal adhesion assembly. Eur. J. Cell Biol..

[7] Park J., Bauer S., Von Der Mark K., Schmuki P. (2007). Nanosize and vitality: TiO_2_ nanotube diameter directs cell fate. Nano Lett..

[8] Park J., Bauer S., Schmuki P., Mark K. (2009). Von Der Narrow Window in Nanoscale Dependent Activation of Endothelial Cell Growth and Differentiation on TiO_2_ Nanotube Surfaces 2009. Nano Lett..

[9] Kulkarni M., Mazare A., Gongadze E., Perutkova Š., Kralj-Iglič V., Milošev I., Schmuki P., Iglič A., Mozetič M. (2015). Titanium nanostructures for biomedical applications. Nanotechnology.

[10] Bauer S., Schmuki P., von der Mark K., Park J. (2012). Engineering biocompatible implant surfaces. Part I: Materials and surfaces. Prog. Mater. Sci..

[11] Mas-Moruno C., Espanol M., Montufar E.B., Mestres G., Aparicio C., Gil F.J., Ginebra M.P. (2013). Bioactive Ceramic and Metallic Surfaces for Bone Engineering. Biomaterials Surface Science.

[12] Xue T., Attarilar S., Liu S., Liu J., Song X., Li L., Zhao B., Tang Y. (2020). Surface modification techniques of titanium and its alloys to functionally optimize their biomedical properties: Thematic review. Front. Bioeng. Biotechnol..

[13] Kurup A., Dhatrak P., Khasnis N. (2020). Surface modification techniques of titanium and titanium alloys for biomedical dental applications: A review. Mater. Today Proc..

[14] Ion R., Necula M.G., Mazare A., Mitran V., Neacsu P., Schmuki P., Cimpean A. (2020). Drug Delivery Systems Based on Titania Nanotubes and Active Agents for Enhanced Osseointegration of Bone Implants. Curr. Med. Chem..

[15] Junkar I., Kulkarni M., Humpolíček P., Capáková Z., Burja B., Mazare A., Schmuki P., Mrak-Poljšak K., Flašker A., Žigon P. (2017). Could Titanium Dioxide Nanotubes Represent a Viable Support System for Appropriate Cells in Vascular Implants?. Advances in Biomembranes and Lipid Self-Assembly.

[16] Ion R., Stoian A.B., Dumitriu C., Grigorescu S., Mazare A., Cimpean A., Demetrescu I., Schmuki P. (2015). Nanochannels formed on TiZr alloy improve biological response. Acta Biomater..

[17] Neacsu P., Mazare A., Schmuki P., Cimpean A. (2015). Attenuation of the macrophage inflammatory activity by TiO_2_ nanotubes via inhibition of MAPK and NF-κB pathways. Int. J. Nanomed..

[18] Salamanna F., Gambardella A., Contartese D., Visani A., Fini M. (2021). Nano-based biomaterials as drug delivery systems against osteoporosis: A systematic review of preclinical and clinical evidence. Nanomaterials.

[19] Negrescu A.M., Cimpean A. (2021). The state of the art and prospects for osteoimmunomodulatory biomaterials. Materials.

[20] Neacsu P., Mazare A., Cimpean A., Park J., Costache M., Schmuki P., Demetrescu I. (2014). Reduced inflammatory activity of RAW 264.7 macrophages on titania nanotube modified Ti surface. Int. J. Biochem. Cell Biol..

[21] Chamberlain L.M., Brammer K.S., Johnston G.W., Chien S., Jin S. (2011). Macrophage Inflammatory Response to TiO_2_ Nanotube Surfaces. J. Biomater. Nanobiotechnol..

[22] Souza J.C.M., Sordi M.B., Kanazawa M., Ravindran S., Henriques B., Silva F.S., Aparicio C., Cooper L.F. (2019). Nano-scale modification of titanium implant surfaces to enhance osseointegration. Acta Biomater..

[23] Takeuchi M., Abe Y., Yoshida Y., Nakayama Y., Okazaki M., Akagawa Y. (2003). Acid pretreatment of titanium implants. Biomaterials.

[24] John A.A., Jaganathan S.K., Supriyanto E., Manikandan A. (2016). Surface modification of titanium and its alloys for the enhancement of osseointegration in orthopaedics. Curr. Sci..

[25] Karthega M., Rajendran N. (2010). Hydrogen peroxide treatment on Ti-6Al-4V alloy: A promising surface modification technique for orthopaedic application. Appl. Surf. Sci..

[26] Janson O., Gururaj S., Pujari-Palmer S., Karlsson Ott M., Strømme M., Engqvist H., Welch K. (2019). Titanium surface modification to enhance antibacterial and bioactive properties while retaining biocompatibility. Mater. Sci. Eng. C.

[27] Wang X., Li B., Zhou L., Ma J., Zhang X., Li H., Liang C., Liu S., Wang H. (2018). Influence of surface structures on biocompatibility of TiO_2_/HA coatings prepared by MAO. Mater. Chem. Phys..

[28] Zhang X., Lv Y., Fu S., Wu Y., Lu X., Yang L., Liu H., Dong Z. (2020). Synthesis, microstructure, anti-corrosion property and biological performances of Mn-incorporated Ca-P/TiO_2_ composite coating fabricated via micro-arc oxidation. Mater. Sci. Eng. C.

[29] Du Q., Wei D., Cheng S., Wang Y., Li B., Jia D., Zhou Y. (2021). Rapid structural evolution and bone inducing mechanism of the multilayer coating with silicon-doped hydroxyapatite crystals on the microwave water steaming-hydrothermally treated titania coating. Appl. Surf. Sci..

[30] Ansar E.B., Ravikumar K., Suresh Babu S., Fernandez F.B., Komath M., Basu B., Harikrishna Varma P.R. (2019). Inducing apatite pre-layer on titanium surface through hydrothermal processing for osseointegration. Mater. Sci. Eng. C.

[31] Kim S., Park C., Cheon K.H., Jung H.-D., Song J., Kim H.E., Jang T.S. (2018). Antibacterial and bioactive properties of stabilized silver on titanium with a nanostructured surface for dental applications. Appl. Surf. Sci..

[32] Mazare A., Anghel A., Surdu-Bob C., Totea G., Demetrescu I., Ionita D. (2018). Silver doped diamond-like carbon antibacterial and corrosion resistance coatings on titanium. Thin Solid Film..

[33] Ţălu Ş., Astinchap B., Abdolghaderi S., Shafiekhani A., Morozov I.A. (2020). Multifractal investigation of Ag/DLC nanocomposite thin films. Sci. Rep..

[34] Lee K., Mazare A., Schmuki P. (2014). One-dimensional titanium dioxide nanomaterials: Nanotubes. Chem. Rev..

[35] Park J., Bauer S., Pittrof A., Killian M.S., Schmuki P., von der Mark K. (2012). Synergistic control of mesenchymal stem cell differentiation by nanoscale surface geometry and immobilized growth factors on TiO_2_ nanotubes. Small.

[36] Ionita D., Bajenaru-Georgescu D., Totea G., Mazare A., Schmuki P., Demetrescu I. (2017). Activity of vancomycin release from bioinspired coatings of hydroxyapatite or TiO_2_ nanotubes. Int. J. Pharm..

[37] Jang I., Choi D.S., Lee J.K., Kim W.T., Cha B.K., Choi W.Y. (2017). Effect of drug-loaded TiO_2_ nanotube arrays on osseointegration in an orthodontic miniscrew: An in-vivo pilot study. Biomed. Microdevices.

[38] Lee J.K., Choi D.S., Jang I., Choi W.Y. (2015). Improved osseointegration of dental titanium implants by TiO_2_ nanotube arrays with recombinant human bone morphogenetic protein-2: A pilot in vivo study. Int. J. Nanomed..

[39] de Stefani A., Bruno G., Preo G., Gracco A. (2020). Application of nanotechnology in orthodontic materials: A state-of-the-art review. Dent. J..

[40] Zinger O., Anselme K., Denzer A., Habersetzer P., Wieland M., Jeanfils J., Hardouin P., Landolt D. (2004). Time-dependent morphology and adhesion of osteoblastic cells on titanium model surfaces featuring scale-resolved topography. Biomaterials.

[41] Lee S.W., Lee M.H., Oh N., Park J.A., Leesungbok R., Ahn S.J. (2012). Correlation between surface hydrophilicity and osteoblastic differentiation on microgrooved titanium substrata. J. Oral Implantol..

[42] Webster T.J., Ejiofor J.U. (2004). Increased osteoblast adhesion on nanophase metals: Ti, Ti6Al4V, and CoCrMo. Biomaterials.

[43] Park J., Bauer S., Schlegel K.A., Neukam F.W., Von Der Mark K., Schmuki P. (2009). TiO_2_ nanotube surfaces: 15 nm—An optimal length scale of surface topography for cell adhesion and differentiation. Small.

[44] Roy P., Berger S., Schmuki P. (2011). TiO_2_ nanotubes: Synthesis and applications. Angew. Chem. Int. Ed..

[45] Zhou X., Nguyen N.T., Ozkan S., Schmuki P. (2014). Anodic TiO_2_ nanotube layers: Why does self-organized growth occur—A mini review. Electrochem. Commun..

[46] Kim D., Lee K., Roy P., Birajdar B.I., Spiecker E., Schmuki P. (2009). Formation of a non-thickness-limited titanium dioxide mesosponge and its use in dye-sensitized solar cells. Angew. Chem. Int. Ed..

[47] Lee K., Kim D., Roy P., Paramasivam I., Birajdar B.I., Spiecker E., Schmuki P. (2010). Anodic formation of thick anatase TiO_2_mesosponge layers for high efficiency photocatalysis. Mater. Sci..

[48] Paramasivam I., Macak J.M., Ghicov A., Schmuki P. (2007). Enhanced photochromism of Ag loaded self-organized TiO_2_ nanotube layers. Chem. Phys. Lett..

[49] Mazare A., Paramasivam I., Schmidt-Stein F., Lee K., Demetrescu I., Schmuki P. (2012). Flame annealing effects on self-organized TiO_2_ nanotubes. Electrochim. Acta.

[50] Albu S.P., Ghicov A., Macak J.M., Schmuki P. (2007). 250 μm long anodic TiO_2_ nanotubes with hexagonal self-ordering. Phys. Status Solidi Rapid Res. Lett..

[51] Albu S.P., Schmuki P. (2010). Highly defined and ordered top-openings in TiO_2_ nanotube arrays. Phys. Status Solidi Rapid Res. Lett..

[52] So S., Lee K., Schmuki P. (2012). Ultrafast growth of highly ordered anodic TiO_2_ nanotubes in lactic acid electrolytes. J. Am. Chem. Soc..

[53] So S., Hwang I., Riboni F., Yoo J.E., Schmuki P. (2016). Robust free standing flow-through TiO_2_ nanotube membranes of pure anatase. Electrochem. Commun..

[54] Mohammadpour F., Behzadi F., Moradi M. (2015). Fast anodically growth of long, small diameter TiO_2_ nanotubes by electropolishing of Ti foils in an ethanol-containing solution. Mater. Lett..

[55] Kulkarni M., Mazare A., Park J., Gongadze E., Killian M.S., Kralj S., von der Mark K., Iglič A., Schmuki P. (2016). Protein interactions with layers of TiO_2_ nanotube and nanopore arrays: Morphology and surface charge influence. Acta Biomater..

[56] Tesler A.B., Altomare M., Schmuki P. (2020). Morphology and Optical Properties of Highly Ordered TiO_2_ Nanotubes Grown in NH_4_F/ o-H_3_PO_4_ Electrolytes in View of Light-Harvesting and Catalytic Applications. ACS Appl. Nano Mater..

[57] Özkan S., Nguyen N.T., Mazare A., Schmuki P. (2016). Controlled spacing of self-organized anodic TiO_2_ nanotubes. Electrochem. Commun..

[58] Kanta A.F., Poelman M., Decroly A. (2015). Electrochemical characterisation of TiO_2_ nanotube array photoanodes for dye-sensitized solar cell application. Sol. Energy Mater. Sol. Cells.

[59] Tang Y.X., Tao J., Zhang Y.Y., Wu T., Tao H.J., Bao Z.G. (2008). Preparation and characterization of TiO_2_ nanotube arrays via anodization of titanium films deposited on FTO conducting glass at room temperature. Wuli Huaxue Xuebao/Acta Phys. Chim. Sin..

[60] Ali G., Chen C., Yoo S.H., Kum J.M., Cho S.O. (2011). Fabrication of complete titania nanoporous structures via electrochemical anodization of Ti. Nanoscale Res. Lett..

[61] Wei W., Berger S., Hauser C., Meyer K., Yang M., Schmuki P. (2010). Transition of TiO_2_ nanotubes to nanopores for electrolytes with very low water contents. Electrochem. Commun..

[62] Berger S., Hahn R., Roy P., Schmuki P. (2010). Self-organized TiO_2_ nanotubes: Factors affecting their morphology and properties. Phys. Status Solidi Basic Res..

[63] Kulkarni M., Mazare A., Schmuki P., Iglic A. (2016). Influence of anodization parameters on morphology of TiO_2_ nanostructured surfaces. Adv. Mater. Lett..

[64] Yoo J.E., Lee K., Altomare M., Selli E., Schmuki P. (2013). Self-organized arrays of single-metal catalyst particles in TiO_2_ cavities: A highly efficient photocatalytic system. Angew. Chem. Int. Ed..

[65] Yoo J.E., Lee K., Schmuki P. (2013). Dewetted Au films form a highly active photocatalytic system on TiO_2_ nanotube-stumps. Electrochem. Commun..

[66] Albu S.P., Schmuki P. (2010). TiO_2_ nanotubes grown in different organic electrolytes: Two-size self-organization, single vs. double-walled tubes, and giant diameters. Phys. Status Solidi Rapid Res. Lett..

[67] Nguyen N.T., Ozkan S., Hwang I., Mazare A., Schmuki P. (2016). TiO_2_ nanotubes with laterally spaced ordering enable optimized hierarchical structures with significantly enhanced photocatalytic H_2_ generation. Nanoscale.

[68] Ozkan S., Nguyen N.T., Mazare A., Hahn R., Cerri I., Schmuki P. (2017). Fast growth of TiO_2_ nanotube arrays with controlled tube spacing based on a self-ordering process at two different scales. Electrochem. Commun..

[69] Albu S.P., Roy P., Virtanen S., Schmuki P. (2010). Self-organized TiO_2_ nanotube arrays: Critical effects on morphology and growth. Isr. J. Chem..

[70] So S., Hwang I., Schmuki P. (2015). Hierarchical DSSC structures based on “single walled” TiO_2_ nanotube arrays reach a back-side illumination solar light conversion efficiency of 8%. Energy Environ. Sci..

[71] Mirabolghasemi H., Liu N., Lee K., Schmuki P. (2013). Formation of ‘single walled’ TiO_2_ nanotubes with significantly enhanced electronic properties for higher efficiency dye-sensitized solar cells. Chem. Commun..

[72] Srivastava S.K., Ghosh Pal B. (2018). Metallic Biomaterials For Dental Implant Systems.

[73] Li Q., Ma G., Li J., Niinomi M., Nakai M., Koizumi Y., Wei D.X., Kakeshita T., Nakano T., Chiba A. (2019). Development of low-Young’s modulus Ti–Nb-based alloys with Cr addition. J. Mater. Sci..

[74] Macak J.M., Tsuchiya H., Taveira L., Ghicov A., Schmuki P. (2005). Self-organized nanotubular oxide layers on Ti-6Al-7Nb and Ti-6Al-4V formed by anodization in NH4F solutions. J. Biomed. Mater. Res. Part A.

[75] Hu N., Hu T., Gao A., Gao N., Starink M.J., Chen Y., Sun W., Liao Q., Tong L., Xu X. (2019). Homogeneous Anodic TiO_2_ Nanotube Layers on Ti–6Al–4V Alloy with Improved Adhesion Strength and Corrosion Resistance. Adv. Mater. Interfaces.

[76] Mazare A., Dilea M., Ionita D., Demetrescu I. (2014). Electrochemical behavior in simulated body fluid of TiO_2_ nanotubes on TiAlNb alloy elaborated in various anodizing electrolyte. Surf. Interface Anal..

[77] Yasuda K., Schmuki P. (2007). Control of morphology and composition of self-organized zirconium titanate nanotubes formed in (NH4)2SO4/NH4F electrolytes. Electrochim. Acta.

[78] Grigorescu S., Pruna V., Titorencu I., Jinga V.V., Mazare A., Schmuki P., Demetrescu I. (2014). The two step nanotube formation on TiZr as scaffolds for cell growth. Bioelectrochemistry.

[79] López-Pavón L., Dagnino-Acosta D., López-Cuéllar E., Meléndez-Anzures F., Zárate-Triviño D., Barrón-González M., Moreno-Cortez I., Kim H.Y., Miyazaki S. (2021). Synthesis of nanotubular oxide on Ti–24Zr–10Nb–2Sn as a drug-releasing system to prevent the growth of Staphylococcus aureus. Chem. Pap..

[80] Ossowska A., Sobieszczyk S., Supernak M., Zielinski A. (2014). Morphology and properties of nanotubular oxide layer on the “Ti-13Zr-13Nb” alloy. Surf. Coat. Technol..

[81] Pérez D.A.G., Jorge Junior A.M., Asato G.H., Lepretre J.C., Roche V., Bolfarini C., Botta W.J. (2019). Surface anodization of the biphasic Ti13Nb13Zr biocompatible alloy: Influence of phases on the formation of TiO_2_ nanostructures. J. Alloy. Compd..

[82] Feng X.J., Macak J.M., Albu S.P., Schmuki P. (2008). Electrochemical formation of self-organized anodic nanotube coating on Ti-28Zr-8Nb biomedical alloy surface. Acta Biomater..

[83] Shrestha N.K., Nah Y.-C., Tsuchiya H., Schmuki P. (2009). Self-organized nano-tubes of TiO_2_-MoO_3_ with enhanced electrochromic properties. Chem. Commun..

[84] Oliveira N.T.C., Verdério J.F., Bolfarini C. (2013). Obtaining self-organized nanotubes on biomedical Ti-Mo alloys. Electrochem. Commun..

[85] Feng X., Macak J.M., Schmuki P. (2007). Flexible self-organization of two size-scales oxide nanotubes on Ti45Nb alloy. Electrochem. Commun..

[86] Ghicov A., Aldabergenova S., Tsuchyia H., Schmuki P. (2006). TiO_2_-Nb_2_O_5_ nanotubes with electrochemically tunable morphologies. Angew. Chem. Int. Ed..

[87] Kim J.J., Byeon I.S., Brantley W.A., Choe H.C. (2015). Highly ordered nanotubular film formation on Ti-25Nb-xZr and Ti-25Ta-xHf. Thin Solid Film..

[88] Jeong Y.H., Kim E.J., Brantley W.A., Choe H.C. (2014). Morphology of hydroxyapatite nanoparticles in coatings on nanotube-formed Ti-Nb-Zr alloys for dental implants. Vacuum.

[89] Jeong Y.H., Kim W.G., Choe H.C., Brantley W.A. (2014). Control of nanotube shape and morphology on Ti-Nb(Ta)-Zr alloys by varying anodizing potential. Thin Solid Film..

[90] Jeong Y.H., Choe H.C., Brantley W.A. (2012). Electrochemical and surface behavior of hydyroxyapatite/Ti film on nanotubular Ti-35Nb-xZr alloys. Appl. Surf. Sci..

[91] Saji V.S., Choe H.C., Brantley W.A. (2009). An electrochemical study on self-ordered nanoporous and nanotubular oxide on Ti-35Nb-5Ta-7Zr alloy for biomedical applications. Acta Biomater..

[92] Hao Y., Li S., Han X., Hao Y., Ai H. (2013). Effects of the surface characteristics of nanoporous titanium oxide films on Ti-24Nb-4Zr-8Sn alloy on the initial adhesion of osteoblast-like MG-63 cells. Exp. Ther. Med..

[93] Kim H.J., Choe H.C. (2020). Magnesium, silicon, and hydroxyapatite deposition on the Ti-xNb-2Ag-2Pt alloy by co-sputtering after nanotube formation. Surf. Coat. Technol..

[94] Jha H., Hahn R., Schmuki P. (2010). Ultrafast oxide nanotube formation on TiNb, TiZr and TiTa alloys by rapid breakdown anodization. Electrochim. Acta.

[95] Hang R., Liu Y., Liu S., Bai L., Gao A., Zhang X., Huang X., Tang B., Chu P.K. (2016). Size-dependent corrosion behavior and cytocompatibility of Ni-Ti-O nanotubes prepared by anodization of biomedical NiTi alloy. Corros. Sci..

[96] Hang R., Huang X., Tian L., He Z., Tang B. (2012). Preparation, characterization, corrosion behavior and bioactivity of Ni_2_O_3_-doped TiO_2_ nanotubes on NiTi alloy. Electrochim. Acta.

[97] Choe H.C. (2011). Nanotubular surface and morphology of Ti-binary and Ti-ternary alloys for biocompatibility. Thin Solid Film..

[98] Jarosz M., Grudzień J., Kapusta-Kołodziej J., Chudecka A., Sołtys M., Sulka G.D. (2020). Anodization of Titanium Alloys for Biomedical Applications. Nanostructured Anodic Metal Oxides: Synthesis and Applications.

[99] Sarraf M., Nasiri-Tabrizi B., Yeong C.H., Madaah Hosseini H.R., Saber-Samandari S., Basirun W.J., Tsuzuki T. (2021). Mixed oxide nanotubes in nanomedicine: A dead-end or a bridge to the future?. Ceram. Int..

[100] Michalska-Domanska M., Łazinska M., Łukasiewicz J., Mol J.M.C., Durejko T. (2020). Self-Organized Anodic Oxides on Titanium Alloys Prepared from Glycol- and Glycerol-Based Electrolytes. Materials.

[101] Gongadze E., Kabaso D., Bauer S., Slivnik T., Schmuki P., van Rienen U., Iglič A. (2011). Adhesion of osteoblasts to a nanorough titanium implant surface. Int. J. Nanomed..

[102] Zhang Y., Andrukhov O., Berner S., Matejka M., Wieland M., Rausch-Fan X., Schedle A. (2010). Osteogenic properties of hydrophilic and hydrophobic titanium surfaces evaluated with osteoblast-like cells (MG63) in coculture with human umbilical vein endothelial cells (HUVEC). Dent. Mater..

[103] Tighineanu A., Ruff T., Albu S., Hahn R., Schmuki P. (2010). Conductivity of TiO_2_ nanotubes: Influence of annealing time and temperature. Chem. Phys. Lett..

[104] Albu S.P., Tsuchiya H., Fujimoto S., Schmuki P. (2010). TiO_2_ nanotubes—Annealing effects on detailed morphology and structure. Eur. J. Inorg. Chem..

[105] Albu S.P., Ghicov A., Aldabergenova S., Drechsel P., LeClere D., Thompson G.E., Macak J.M., Schmuki P. (2008). Formation of double-walled TiO_2_ nanotubes and robust anatase membranes. Adv. Mater..

[106] Regonini D., Jaroenworaluck A., Stevens R., Bowen C.R. (2010). Effect of heat treatment on the properties and structure of TiO_2_ nanotubes: Phase composition and chemical composition. Surf. Interface Anal..

[107] Mazare A., Paramasivam I., Lee K., Schmuki P. (2011). Improved water-splitting behaviour of flame annealed TiO_2_ nanotubes. Electrochem. Commun..

[108] Tighineanu A., Albu S.P., Schmuki P. (2014). Conductivity of anodic TiO_2_ nanotubes: Influence of annealing conditions. Phys. Status Solidi Rapid Res. Lett..

[109] Jarosz M., Syrek K., Kapusta-Kołodziej J., Mech J., Małek K., Hnida K., Łojewski T., Jaskuła M., Sulka G.D. (2015). Heat treatment effect on crystalline structure and photoelectrochemical properties of anodic TiO_2_ nanotube arrays formed in ethylene glycol and glycerol based electrolytes. J. Phys. Chem. C.

[110] Kowalski D., Kim D., Schmuki P. (2013). TiO_2_ nanotubes, nanochannels and mesosponge: Self-organized formation and applications. Nano Today.

[111] Mazare A., Totea G., Burnei C., Schmuki P., Demetrescu I., Ionita D. (2016). Corrosion, antibacterial activity and haemocompatibility of TiO_2_ nanotubes as a function of their annealing temperature. Corros. Sci..

[112] Pegueroles M., Aparicio C., Bosio M., Engel E., Gil F.J., Planell J.A., Altankov G. (2010). Spatial organization of osteoblast fibronectin matrix on titanium surfaces: Effects of roughness, chemical heterogeneity and surface energy. Acta Biomater..

[113] Cochran D.L., Schenk R.K., Lussi A., Higginbottom F.L., Buser D. (1998). Bone response to unloaded and loaded titanium implants with a sandblasted and acid-etched surface: A histometric study in the canine mandible. J. Biomed. Mater. Res..

[114] Ivanoff C.J., Hallgren C., Widmark G., Sennerby L., Wennerberg A. (2001). Histologic evaluation of the bone integration of TiO_2_ blasted and turned titanium microimplants in humans. Clin. Oral Implant. Res..

[115] Matos G.R.M. (2021). Surface Roughness of Dental Implant and Osseointegration. J. Maxillofac. Oral Surg..

[116] Ueno T., Yamada M., Suzuki T., Minamikawa H., Sato N., Hori N., Takeuchi K., Hattori M., Ogawa T. (2010). Enhancement of bone-titanium integration profile with UV-photofunctionalized titanium in a gap healing model. Biomaterials.

[117] Siqueira R., Ferreira J.A., Rizzante F.A.P., Moura G.F., Mendonça D.B.S., de Magalhães D., Cimões R., Mendonça G. (2021). Hydrophilic titanium surface modulates early stages of osseointegration in osteoporosis. J. Periodontal Res..

[118] Sugita Y., Saruta J., Taniyama T., Kitajima H., Hirota M., Ikeda T., Ogawa T. (2020). UV-pre-treated and protein-adsorbed titanium implants exhibit enhanced osteoconductivity. Int. J. Mol. Sci..

[119] Anitha V.C., Menon D., Nair S.V., Prasanth R. (2010). Electrochemical tuning of titania nanotube morphology in inhibitor electrolytes. Electrochim. Acta.

[120] Kontos A.G., Kontos A.I., Tsoukleris D.S., Likodimos V., Kunze J., Schmuki P., Falaras P. (2009). Photo-induced effects on self-organized TiO_2_ nanotube arrays: The influence of surface morphology. Nanotechnology.

[121] Vardaki M., Mohajernia S., Pantazi A., Nica I.C., Enachescu M., Mazare A., Demetrescu I., Schmuki P. (2019). Post treatments effect on TiZr nanostructures fabricated via anodizing. J. Mater. Res. Technol..

[122] Wang G., Feng H., Jin W., Gao A., Peng X., Li W., Wu H., Li Z., Chu P.K. (2017). Long-term antibacterial characteristics and cytocompatibility of titania nanotubes loaded with Au nanoparticles without photocatalytic effects. Appl. Surf. Sci..

[123] Durdu S., Cihan G., Yalcin E., Altinkok A. (2021). Characterization and mechanical properties of TiO_2_ nanotubes formed on titanium by anodic oxidation. Ceram. Int..

[124] Wu S., Shen X., Chen M., Yie K.H.R., Zhou Z., Al-Baadani M.A., Fang K., Al-Bishari A.M., Deng Z., Liu J. (2021). Multifunctional TaCu-Nanotubes Coated Titanium for Enhanced Bacteriostatic, Angiogenic and Osteogenic Properties.

[125] Lai M., Yan X., Shen K., Tang Q., Fang X., Zhang C., Zhu Z., Hou Y. (2020). The effect of calcitonin gene-related peptide functionalized TiO_2_ nanotubes on osteoblast and osteoclast differentiation in vitro. Colloids Surf. A Physicochem. Eng. Asp..

[126] Mei S., Wang H., Wang W., Tong L., Pan H., Ruan C., Ma Q., Liu M., Yang H., Zhang L. (2014). Antibacterial effects and biocompatibility of titanium surfaces with graded silver incorporation in titania nanotubes. Biomaterials.

[127] Jarosz M., Pawlik A., Szuwarzyński M., Jaskuła M., Sulka G.D. (2016). Nanoporous anodic titanium dioxide layers as potential drug delivery systems: Drug release kinetics and mechanism. Colloids Surf. B Biointerfaces.

[128] Peng Z., Ni J. (2019). Surface properties and bioactivity of TiO_2_ nanotube array prepared by two-step anodic oxidation for biomedical applications. R. Soc. Open Sci..

[129] Huhtamäki T., Tian X., Korhonen J.T., Ras R.H.A. (2018). Surface-wetting characterization using contact-angle measurements. Nat. Protoc..

[130] Parvate S., Dixit P., Chattopadhyay S. (2020). Superhydrophobic Surfaces: Insights from Theory and Experiment. J. Phys. Chem. B.

[131] Vogler E.A. (1998). Structure and reactivity of water at biomaterial surfaces. Adv. Colloid Interface Sci..

[132] Gentleman M.M., Gentleman E. (2014). The role of surface free energy in osteoblast-biomaterial interactions. Int. Mater. Rev..

[133] Song W., Mano J.F. (2013). Interactions between cells or proteins and surfaces exhibiting extreme wettabilities. Soft Matter.

[134] De Jonge L.T., Leeuwenburgh S.C.G., Wolke J.G.C., Jansen J.A. (2008). Organic-inorganic surface modifications for titanium implant surfaces. Pharm. Res..

[135] Balaur E., Macak J.M., Tsuchiya H., Schmuki P. (2005). Wetting behaviour of layers of TiO_2_ nanotubes with different diameters. J. Mater. Chem..

[136] Tesler A.B., Prado L.H., Khusniyarov M.M., Thievessen I., Mazare A., Fischer L., Virtanen S., Goldmann W.H., Schmuki P. (2021). A One-Pot Universal Approach to Fabricate Lubricant-Infused Slippery Surfaces on Solid Substrates. Adv. Funct. Mater..

[137] Li X.M., Reinhoudt D., Crego-Calama M. (2007). What do we need for a superhydrophobic surface? A review on the recent progress in the preparation of superhydrophobic surfaces. Chem. Soc. Rev..

[138] Zhang Y.L., Xia H., Kim E., Sun H.B. (2012). Recent developments in superhydrophobic surfaces with unique structural and functional properties. Soft Matter.

[139] Eliaz N. (2019). Corrosion of metallic biomaterials: A review. Materials.

[140] Seah K.H.W., Thampuran R., Teoh S.H. (1998). The influence of pore morphology on corrosion. Corros. Sci..

[141] Demetrescu I., Pirvu C., Mitran V. (2010). Effect of nano-topographical features of Ti/TiO_2_ electrode surface on cell response and electrochemical stability in artificial saliva. Bioelectrochemistry.

[142] Liu C., Wang Y., Wang M., Huang W., Chu P.K. (2011). Electrochemical stability of TiO_2_ nanotubes with different diameters in artificial saliva. Surf. Coat. Technol..

[143] Man I., Pirvu C., Demetrescu I. (2008). Enhancing titanium stability in Fusayama saliva using electrochemical elaboration of TiO_2_ nanotubes. Rev. Chim..

[144] Yu W.Q., Qiu J., Xu L., Zhang F.Q. (2009). Corrosion behaviors of TiO_2_ nanotube layers on titanium in Hank’s solution. Biomed. Mater..

[145] Ali Yahia S.A., Hamadou L., Kadri A., Benbrahim N., Sutter E.M.M. (2012). Effect of Anodizing Potential on the Formation and EIS Characteristics of TiO_2_ Nanotube Arrays. J. Electrochem. Soc..

[146] Monetta T., Acquesta A., Carangelo A., Bellucci F. (2017). TiO_2_ nanotubes on Ti dental implant. Part 2: EIS characterization in Hank’s solution. Metals.

[147] Acquesta A., Carangelo A., Monetta T. (2018). TiO_2_ Nanotubes on Ti dental implant. Part 3: Electrochemical behavior in hank’s solution of titania nanotubes formed in ethylene glycol. Metals.

[148] Al-Saady F.A.A., Rushdi S.A., Abbar A.H. (2020). Improvement the corrosion Behavior of Titanium by Nanotubular Oxide in a simulated saliva solution. IOP Conf. Ser. Mater. Sci. Eng..

[149] Indira K., Mudali U.K., Rajendran N. (2014). In-vitro biocompatibility and corrosion resistance of strontium incorporated TiO_2_ nanotube arrays for orthopaedic applications. J. Biomater. Appl..

[150] Mazare A., Ionita D., Totea G., Demetrescu I. (2014). Calcination condition effect on microstructure, electrochemical and hemolytic behavior of amorphous nanotubes on Ti6Al7Nb alloy. Surf. Coat. Technol..

[151] Mohan L., Anandan C., Rajendran N. (2015). Electrochemical behavior and effect of heat treatment on morphology, crystalline structure of self-organized TiO_2_ nanotube arrays on Ti–6Al–7Nb for biomedical applications. Mater. Sci. Eng. C.

[152] Kim W.G., Choe H.C. (2009). Nanostructure and corrosion behaviors of nanotube formed Ti-Zr alloy. Trans. Nonferrous Met. Soc. China.

[153] Indira K., Kamachi Mudali U., Rajendran N. (2013). Corrosion behavior of electrochemically assembled nanoporous titania for biomedical applications. Ceram. Int..

[154] Mazǎre A., Voicu G., Truscǎ R., Ioniţǎ D. (2011). Heat treatment of TiO_2_ nanotubes, a way to significantly change their behaviour. UPB Sci. Bull. Ser. B Chem. Mater. Sci..

[155] Kelly R.G., Scully J.R., Shoesmith D., Buchheit R.G. (2002). Electrochemical Techniques in Corrosion Science and Engineering.

[156] Kear G., Walsh F.C. (2005). The characteristics of a true tafel slope. Corros. Mater..

[157] Burstein G.T. (2005). A hundred years of Tafel’s Equation: 1905-2005. Corros. Sci..

[158] Buchanan R.A., Stansbury E.E. (2013). Electrochemical Corrosion. Handbook of Environmental Degradation of Materials.

[159] Al-Mobarak N.A., Al-Swayih A.A. (2014). Development of titanium surgery implants for improving osseointegration through formation of a titanium nanotube layer. Int. J. Electrochem. Sci..

[160] Sun Y., Rong Y., Zhao Y., Zhao Y., Hang R., Yao X., Chu P.K. (2021). Electrochemical stability, corrosion behavior, and biological properties of Ni–Ti–O nanoporous layers anodically on NiTi alloy. Corros. Sci..

[161] Barjaktarević D.R., Djokić V.R., Bajat J.B., Dimić I.D., Cvijović-Alagić I.L., Rakin M.P. (2019). The influence of the surface nanostructured modification on the corrosion resistance of the ultrafine-grained Ti–13Nb–13Zr alloy in artificial saliva. Theor. Appl. Fract. Mech..

[162] Lauffenburger D.A., Horwitz A.F. (1996). Cell migration: A physically integrated molecular process. Cell.

[163] Von Der Mark K., Park J., Bauer S., Schmuki P. (2010). Nanoscale engineering of biomimetic surfaces: Cues from the extracellular matrix. Cell Tissue Res..

[164] von der Mark K., Park J. (2013). Engineering biocompatible implant surfaces: Part II: Cellular recognition of biomaterial surfaces: Lessons from cell–matrix interactions. Prog. Mater. Sci..

[165] Huang S., Chen C.S., Ingber D.E. (1998). Control of cyclin D1, p27(Kip1), and cell cycle progression in human capillary endothelial cells by cell shape and cytoskeletal tension. Mol. Biol. Cell.

[166] Mendonça G., Mendonça D.B.S., Aragão F.J.L., Cooper L.F. (2008). Advancing dental implant surface technology—From micron- to nanotopography. Biomaterials.

[167] Dalby M.J., Riehle M.O., Yarwood S.J., Wilkinson C.D.W., Curtis A.S.G. (2003). Nucleus alignment and cell signaling in fibroblasts: Response to a micro-grooved topography. Exp. Cell Res..

[168] Spatz J.P., Niemeyer C.M., Mirkin C.A. (2004). Cell-Nanostructure Interactions. Nanobiotechnology: Concepts, Applications and Perspectives.

[169] Seifert G., Eschrig E., Bieger W., Porezag D., Seifert G., Widany J., Weich F., Porezag D., Bilek M.M.M., Mckenzie D.R. (2002). Oxidation-Resistant Gold-55 Clusters. Science.

[170] Liu H., Webster T.J. (2007). Nanomedicine for implants: A review of studies and necessary experimental tools. Biomaterials.

[171] Wang N., Li H., Lü W., Li J., Wang J., Zhang Z., Liu Y. (2011). Effects of TiO_2_ nanotubes with different diameters on gene expression and osseointegration of implants in minipigs. Biomaterials.

[172] Bauer S., Park J., Pittrof A., Song Y.-Y., von der Mark K., Schmuki P. (2011). Covalent functionalization of TiO_2_ nanotube arrays with EGF and BMP-2 for modified behavior towards mesenchymal stem cells. Integr. Biol..

[173] Brammer K.S., Oh S., Cobb C.J., Bjursten L.M., van der Heyde H., Jin S. (2009). Improved bone-forming functionality on diameter-controlled TiO_2_ nanotube surface. Acta Biomater..

[174] Khaw J.S., Bowen C.R., Cartmell S.H. (2020). Effect of TiO_2_ nanotube pore diameter on human mesenchymal stem cells and human osteoblasts. Nanomaterials.

[175] Bauer S., Park J., Faltenbacher J., Berger S., von der Mark K., Schmuki P. (2009). Size selective behavior of mesenchymal stem cells on ZrO_2_ and TiO_2_ nanotube arrays. Integr. Biol..

[176] Chang Y., Shao Y., Liu Y., Xia R., Tong Z., Zhang J., Zhai Z., Cheng W., Li H. (2019). Mechanical strain promotes osteogenic differentiation of mesenchymal stem cells on TiO_2_ nanotubes substrate. Biochem. Biophys. Res. Commun..

[177] Tong Z., Liu Y., Xia R., Chang Y., Hu Y., Liu P., Zhai Z., Zhang J., Li H. (2020). F-actin Regulates Osteoblastic Differentiation of Mesenchymal Stem Cells on TiO_2_ Nanotubes Through MKL1 and YAP/TAZ. Nanoscale Res. Lett..

[178] Dobbenga S., Fratila-Apachitei L.E., Zadpoor A.A. (2016). Nanopattern-induced osteogenic differentiation of stem cells—A systematic review. Acta Biomater..

[179] McMurray R.J., Gadegaard N., Tsimbouri P.M., Burgess K.V., McNamara L.E., Tare R., Murawski K., Kingham E., Oreffo R.O.C., Dalby M.J. (2011). Nanoscale surfaces for the long-term maintenance of mesenchymal stem cell phenotype and multipotency. Nat. Mater..

[180] Seo C.H., Jeong H., Feng Y., Montagne K., Ushida T., Suzuki Y., Furukawa K.S. (2014). Micropit surfaces designed for accelerating osteogenic differentiation of murine mesenchymal stem cells via enhancing focal adhesion and actin polymerization. Biomaterials.

[181] Li L., Yang S., Xu L., Li Y., Fu Y., Zhang H., Song J. (2019). Nanotopography on titanium promotes osteogenesis via autophagy-mediated signaling between YAP and β-catenin. Acta Biomater..

[182] Gao Q., Hou Y., Li Z., Hu J., Huo D., Zheng H., Zhang J., Yao X., Gao R., Wu X. (2021). mTORC2 regulates hierarchical micro/nano topography-induced osteogenic differentiation via promoting cell adhesion and cytoskeletal polymerization. J. Cell. Mol. Med..

[183] Wang W., Zhao L., Ma Q., Wang Q., Chu P.K., Zhang Y. (2012). The role of the Wnt/β-catenin pathway in the effect of implant topography on MG63 differentiation. Biomaterials.

[184] Lv L., Liu Y., Zhang P., Bai X., Ma X., Wang Y., Li H., Wang L., Zhou Y. (2018). The epigenetic mechanisms of nanotopography-guided osteogenic differentiation of mesenchymal stem cells via high-throughput transcriptome sequencing. Int. J. Nanomed..

[185] Teo B.K.K., Wong S.T., Lim C.K., Kung T.Y.S., Yap C.H., Ramagopal Y., Romer L.H., Yim E.K.F. (2013). Nanotopography Modulates Mechanotransduction of Stem Cells and Induces Differentiation through Focal Adhesion Kinase. ACS Nano.

[186] Trappmann B., Gautrot J.E., Connelly J.T., Strange D.G.T., Li Y., Oyen M.L., Cohen Stuart M.A., Boehm H., Li B., Vogel V. (2012). Extracellular-matrix tethering regulates stem-cell fate. Nat. Mater..

[187] Aragona M., Panciera T., Manfrin A., Giulitti S., Michielin F., Elvassore N., Dupont S., Piccolo S. (2013). A Mechanical Checkpoint Controls Multicellular Growth through YAP/TAZ Regulation by Actin-Processing Factors. Cell.

[188] Necula M.G., Mazare A., Ion R.N., Ozkan S., Park J., Schmuki P., Cimpean A. (2019). Lateral spacing of TiO_2_ nanotubes modulates osteoblast behavior. Materials.

[189] Smith B.S., Capellato P., Kelley S., Gonzalez-Juarrero M., Popat K.C. (2013). Reduced in vitro immune response on titania nanotube arrays compared to titanium surface. Biomater. Sci..

[190] Yao S., Feng X., Li W., Wang L.N., Wang X. (2017). Regulation of RAW 264.7 macrophages behavior on anodic TiO_2_ nanotubular arrays. Front. Mater. Sci..

[191] Bai L., Zhao Y., Chen P., Zhang X., Huang X., Du Z., Crawford R., Yao X., Tang B., Hang R. (2021). Targeting Early Healing Phase with Titania Nanotube Arrays on Tunable Diameters to Accelerate Bone Regeneration and Osseointegration. Small.

[192] Huang Q., Yang Y., Zheng D., Song R., Zhang Y., Jiang P., Vogler E.A., Lin C. (2017). Effect of construction of TiO_2_ nanotubes on platelet behaviors: Structure-property relationships. Acta Biomater..

[193] Gong Z., Hu Y., Gao F., Quan L., Liu T., Gong T., Pan C. (2019). Effects of diameters and crystals of titanium dioxide nanotube arrays on blood compatibility and endothelial cell behaviors. Colloids Surf. B Biointerfaces.

[194] Bai L., Yang Y., Mendhi J., Du Z., Hao R., Hang R., Yao X., Huang N., Tang B., Xiao Y. (2018). The effects of TiO_2_ nanotube arrays with different diameters on macrophage/endothelial cell response and ex vivo hemocompatibility. J. Mater. Chem. B.

[195] Von Wilmowsky C., Bauer S., Lutz R., Meisel M., Neukam F.W., Toyoshima T., Schmuki P., Nkenke E., Schlegel K.A. (2009). In Vivo Evaluation of Anodic TiO_2_ Nanotubes; An Experimental Study in the Pig. J. Biomed. Mater. Res. Part B Appl. Biomater..

[196] Alves-Rezende M.C.R., Capalbo L.C., De Oliveira Limírio J.P.J., Capalbo B.C., Limírio P.H.J.O., Rosa J.L. (2020). The role of TiO_2_ nanotube surface on osseointegration of titanium implants: Biomechanical and histological study in rats. Microsc. Res. Tech..

[197] Baker E.A., Vara A.D., Salisbury M.R., Fleischer M.M., Baker K.C., Fortin P.T., Roberts R.V., Friedrich C.R. (2020). Titania nanotube morphologies for osseointegration via models of in vitro osseointegrative potential and in vivo intramedullary fixation. J. Biomed. Mater. Res. Part B Appl. Biomater..

[198] Wang F., Li C., Zhang S., Liu H. (2020). Role of TiO_2_ Nanotubes on the Surface of Implants in Osseointegration in Animal Models: A Systematic Review and Meta-Analysis. J. Prosthodont..

[199] Balasundaram G., Yao C., Webster T.J. (2008). TiO_2_ nanotubes functionalized with regions of bone morphogenetic protein-2 increases osteoblast adhesion. J. Biomed. Mater. Res. Part A.

[200] Lai M., Cai K., Zhao L., Chen X., Hou Y., Yang Z. (2011). Surface functionalization of TiO_2_ nanotubes with bone morphogenetic protein 2 and its synergistic effect on the differentiation of mesenchymal stem cells. Biomacromolecules.

[201] Tao B., Deng Y., Song L., Ma W., Qian Y., Lin C., Yuan Z., Lu L., Chen M., Yang X. (2019). BMP2-loaded titania nanotubes coating with pH-responsive multilayers for bacterial infections inhibition and osteogenic activity improvement. Colloids Surf. B Biointerfaces.

[202] Keceli H.G., Bayram C., Celik E., Ercan N., Demirbilek M., Nohutcu R.M. (2020). Dual delivery of platelet-derived growth factor and bone morphogenetic factor-6 on titanium surface to enhance the early period of implant osseointegration. J. Periodontal Res..

[203] Lai M., Jin Z., Su Z. (2017). Surface modification of TiO_2_ nanotubes with osteogenic growth peptide to enhance osteoblast differentiation. Mater. Sci. Eng. C.

[204] Markx G.H. (2008). The use of electric fields in tissue engineering: A review. Organogenesis.

[205] Griffin M., Bayat A. (2011). Electrical stimulation in bone healing: Critical analysis by evaluating levels of evidence. Eplasty.

[206] Hart F.X., Palisano J.R. (2018). The Application of Electric Fields in Biology and Medicine. Electric Field.

[207] Iwasa S.N., Babona-Pilipos R., Morshead C.M. (2017). Environmental Factors That Influence Stem Cell Migration: An “electric Field”. Stem Cells Int..

[208] Thrivikraman G., Boda S.K., Basu B. (2018). Unraveling the mechanistic effects of electric field stimulation towards directing stem cell fate and function: A tissue engineering perspective. Biomaterials.

[209] Mycielska M.E., Djamgoz M.B.A. (2004). Cellular mechanisms of direct-current electric field effects: Galvanotaxis and metastatic disease. J. Cell Sci..

[210] Srirussamee K., Xue R., Mobini S., Cassidy N.J., Cartmell S.H. (2021). Changes in the extracellular microenvironment and osteogenic responses of mesenchymal stem/stromal cells induced by in vitro direct electrical stimulation. J. Tissue Eng..

[211] Stephan M., Zimmermann J., Klinder A., Sahm F., van Rienen U., Kämmerer P.W., Bader R., Jonitz-Heincke A. (2020). Establishment and Evaluation of an In Vitro System for Biophysical Stimulation of Human Osteoblasts. Cells.

[212] Meng S., Rouabhia M., Zhang Z. (2013). Electrical stimulation modulates osteoblast proliferation and bone protein production through heparin-bioactivated conductive scaffolds. Bioelectromagnetics.

[213] Babona-Pilipos R., Liu N., Pritchard-Oh A., Mok A., Badawi D., Popovic M.R., Morshead C.M. (2018). Calcium influx differentially regulates migration velocity and directedness in response to electric field application. Exp. Cell Res..

[214] Naskar S., Kumaran V., Markandeya Y.S., Mehta B., Basu B. (2020). Neurogenesis-on-Chip: Electric field modulated transdifferentiation of human mesenchymal stem cell and mouse muscle precursor cell coculture. Biomaterials.

[215] Cheng Y.C., Chen C.H., Kuo H.W., Yen T.L., Mao Y.Y., Hu W.W. (2019). Electrical stimulation through conductive substrate to enhance osteo-differentiation of human dental pulp-derived stem cells. Appl. Sci..

[216] Jaatinen C. (2017). The Effect of an Applied Electric Current on Cell Proliferation, Viability, Morphology, Adhesion, and Stem Cell Differentiation. Ph.D. Thesis.

[217] Gabi M., Sannomiya T., Larmagnac A., Puttaswamy M., Vörös J. (2009). Influence of applied currents on the viability of cells close to microelectrodes. Integr. Biol..

[218] Yarmush M.L., Golberg A., Serša G., Kotnik T., Miklavčič D. (2014). Electroporation-based technologies for medicine: Principles, applications, and challenges. Annu. Rev. Biomed. Eng..

[219] Grunert P.C., Jonitz-Heincke A., Su Y., Souffrant R., Hansmann D., Ewald H., Krüger A., Mittelmeier W., Bader R. (2014). Establishment of a Novel In Vitro Test Setup for Electric and Magnetic Stimulation of Human Osteoblasts. Cell Biochem. Biophys..

[220] Hiemer B., Ziebart J., Jonitz-Heincke A., Grunert P.C., Su Y., Hansmann D., Bader R. (2016). Magnetically induced electrostimulation of human osteoblasts results in enhanced cell viability and osteogenic differentiation. Int. J. Mol. Med..

[221] Jin G., Kim G. (2013). The effect of sinusoidal AC electric stimulation of 3D PCL/CNT and PCL/β-TCP based bio-composites on cellular activities for bone tissue regeneration. J. Mater. Chem. B.

[222] Zhang J., Li M., Kang E.T., Neoh K.G. (2016). Electrical stimulation of adipose-derived mesenchymal stem cells in conductive scaffolds and the roles of voltage-gated ion channels. Acta Biomater..

[223] Park J., Mazare A., Schneider H., von der Mark K., Fischer M.J.M., Schmuki P. (2016). Electric Field-Induced Osteogenic Differentiation on TiO 2 Nanotubular Layer. Tissue Eng. Part C Methods.

[224] Mazare A., Park J., Simons S., Mohajernia S., Hwang I., Yoo J.E., Schneider H., Fischer M.J., Schmuki P. (2019). Black TiO_2_ nanotubes: Efficient electrodes for triggering electric field-induced stimulation of stem cell growth. Acta Biomater..

[225] Portan D.V., Deligianni D.D., Papanicolaou G.C., Kostopoulos V., Psarras G.C., Tyllianakis M. (2019). Combined Optimized Effect of a Highly Self-Organized Nanosubstrate and an Electric Field on Osteoblast Bone Cells Activity. Biomed Res. Int..

[226] Sahm F., Ziebart J., Jonitz-Heincke A., Hansmann D., Dauben T., Bader R. (2020). Alternating electric fields modify the function of human osteoblasts growing on and in the surroundings of titanium electrodes. Int. J. Mol. Sci..

[227] Gulati K., Kogawa M., Maher S., Atkins G., Findlay D., Losic D., Losic D., Santos A. (2015). Titania nanotubes for local drug delivery from implant surfaces. Electrochemically Engineered Nanoporous Materials. Springer Series in Materials Science.

[228] Maher S., Losic D. (2018). Nanoengineered titania nanotube arrays for localized drug delivery and enhanced osseointegration. Nanotechnologies in Preventive and Regenerative Medicine.

[229] De Santo I., Sanguigno L., Causa F., Monetta T., Netti P.A. (2012). Exploring doxorubicin localization in eluting TiO_2_ nanotube arrays through fluorescence correlation spectroscopy analysis. Analyst.

[230] Moseke C., Hage F., Vorndran E., Gbureck U. (2012). TiO_2_ nanotube arrays deposited on Ti substrate by anodic oxidation and their potential as a long-term drug delivery system for antimicrobial agents. Appl. Surf. Sci..

[231] Wang H., Wei L., Wang Z., Chen S. (2016). Preparation, characterization and long-term antibacterial activity of Ag-poly(dopamine)-TiO_2_ nanotube composites. RSC Adv..

[232] Nemati S.H., Hadjizadeh A. (2017). Gentamicin-Eluting Titanium Dioxide Nanotubes Grown on the Ultrafine-Grained Titanium. AAPS PharmSciTech.

[233] Shirazi-Fard S., Mohammadpour F., Zolghadr A.R., Klein A. (2021). Encapsulation and Release of Doxorubicin from TiO_2_ Nanotubes: Experiment, Density Functional Theory Calculations, and Molecular Dynamics Simulation. J. Phys. Chem. B.

[234] Aw M.S., Gulati K., Losic D. (2011). Controlling Drug Release from Titania Nanotube Arrays Using Polymer Nanocarriers and Biopolymer Coating. J. Biomater. Nanobiotechnol..

[235] Shokuhfar T., Sinha-Ray S., Sukotjo C., Yarin A.L. (2013). Intercalation of anti-inflammatory drug molecules within TiO_2_ nanotubes. RSC Adv..

[236] Pawlik A., Jarosz M., Syrek K., Sulka G.D. (2017). Co-delivery of ibuprofen and gentamicin from nanoporous anodic titanium dioxide layers. Colloids Surf. B Biointerfaces.

[237] Mohan L., Anandan C., Rajendran N. (2016). Drug release characteristics of quercetin-loaded TiO_2_ nanotubes coated with chitosan. Int. J. Biol. Macromol..

[238] Shidfar S., Tavangarian F., Nemati N.H., Fahami A. (2017). Drug delivery behavior of titania nanotube arrays coated with chitosan polymer. Mater. Discov..

[239] Chen X., Cai K., Fang J., Lai M., Hou Y., Li J., Luo Z., Hu Y., Tang L. (2013). Fabrication of selenium-deposited and chitosan-coated titania nanotubes with anticancer and antibacterial properties. Colloids Surf. B Biointerfaces.

[240] Hu Y., Cai K., Luo Z., Xu D., Xie D., Huang Y., Yang W., Liu P. (2012). TiO_2_ nanotubes as drug nanoreservoirs for the regulation of mobility and differentiation of mesenchymal stem cells. Acta Biomater..

[241] Zhao L., Wang H., Huo K., Cui L., Zhang W., Ni H., Zhang Y., Wu Z., Chu P.K. (2011). Antibacterial nano-structured titania coating incorporated with silver nanoparticles. Biomaterials.

[242] Taipina M.O., de Mello M.G., Tamborlin L., Pereira K.D., Luchessi A.D., Cremasco A., Caram R. (2021). A novel Ag doping Ti alloys route: Formation and antibacterial effect of the TiO_2_ nanotubes. Mater. Chem. Phys..

[243] Gunputh U.F., Le H., Lawton K., Besinis A., Tredwin C., Handy R.D. (2020). Antibacterial properties of silver nanoparticles grown in situ and anchored to titanium dioxide nanotubes on titanium implant against Staphylococcus aureus. Nanotoxicology.

[244] Gao A., Hang R., Huang X., Zhao L., Zhang X., Wang L., Tang B., Ma S., Chu P.K. (2014). The effects of titania nanotubes with embedded silver oxide nanoparticles on bacteria and osteoblasts. Biomaterials.

[245] Sarraf M., Dabbagh A., Abdul Razak B., Mahmoodian R., Nasiri-Tabrizi B., Hosseini H.R.M., Saber-Samandari S., Abu Kasim N.H., Abdullah H., Sukiman N.L. (2018). Highly-ordered TiO_2_ nanotubes decorated with Ag_2_O nanoparticles for improved biofunctionality of Ti6Al4V. Surf. Coat. Technol..

[246] Huo K., Zhang X., Wang H., Zhao L., Liu X., Chu P.K. (2013). Osteogenic activity and antibacterial effects on titanium surfaces modified with Zn-incorporated nanotube arrays. Biomaterials.

[247] Roguska A., Belcarz A., Pisarek M., Ginalska G., Lewandowska M. (2015). TiO_2_ nanotube composite layers as delivery system for ZnO and Ag nanoparticles—An unexpected overdose effect decreasing their antibacterial efficacy. Mater. Sci. Eng. C.

[248] Wang X., Qiao J., Yuan F., Hang R., Huang X., Tang B. (2014). In situ growth of self-organized Cu-containing nano-tubes and nano-pores on Ti90-xCu10Alx (x = 0, 45) alloys by one-pot anodization and evaluation of their antimicrobial activity and cytotoxicity. Surf. Coat. Technol..

[249] Xin Y., Jiang J., Huo K., Hu T., Chu P.K. (2009). Bioactive SrTiO_3_ Nanotube Arrays: Osteoporotic Bone Implants. ASC Nano.

[250] Pan C., Liu T., Yang Y., Liu T., Gong Z., Wei Y., Quan L., Yang Z., Liu S. (2020). Incorporation of Sr^2+^ and Ag nanoparticles into TiO_2_ nanotubes to synergistically enhance osteogenic and antibacterial activities for bone repair. Mater. Des..

[251] Jin G., Qin H., Cao H., Qian S., Zhao Y., Peng X., Zhang X., Liu X., Chu P.K. (2014). Synergistic effects of dual Zn/Ag ion implantation in osteogenic activity and antibacterial ability of titanium. Biomaterials.

[252] Li B., Hao J., Min Y., Xin S., Guo L., He F., Liang C., Wang H., Li H. (2015). Biological properties of nanostructured Ti incorporated with Ca, P and Ag by electrochemical method. Mater. Sci. Eng. C.

[253] Roguska A., Pisarek M., Belcarz A., Marcon L., Holdynski M., Andrzejczuk M., Janik-Czachor M. (2016). Improvement of the bio-functional properties of TiO_2_ nanotubes. Appl. Surf. Sci..

[254] Draghi L., Preda V., Moscatelli M., Santin M., Chiesa R. (2020). Gentamicin-Loaded TiO_2_ Nanotubes as Improved Antimicrobial Surfaces for Orthopedic Implants. Front. Mater..

[255] Hashemi A., Ezati M., Mohammadnejad J., Houshmand B., Faghihi S. (2020). Chitosan coating of tio2 nanotube arrays for improved metformin release and osteoblast differentiation. Int. J. Nanomed..

[256] Shen X., Ma P., Hu Y., Xu G., Xu K., Chen W., Ran Q., Dai L., Yu Y., Mu C. (2016). Alendronate-loaded hydroxyapatite-TiO_2_ nanotubes for improved bone formation in osteoporotic rabbits. J. Mater. Chem. B.

[257] Lai M., Jin Z., Yang X., Wang H., Xu K. (2017). The controlled release of simvastatin from TiO_2_ nanotubes to promote osteoblast differentiation and inhibit osteoclast resorption. Appl. Surf. Sci..

[258] Perumal A., Kanumuri R., Rayala S.K., Nallaiyan R. (2020). Fabrication of bioactive corrosion-resistant polyaniline/TiO_2_ nanotubes nanocomposite and their application in orthopedics. J. Mater. Sci..

[259] Coman A.N., Mare A., Tanase C., Bud E., Rusu A. (2021). Silver-Deposited Nanoparticles on the Titanium Nanotubes Surface as a Promising Antibacterial Material into Implants. Metals.

[260] Bonnelye E., Chabadel A., Saltel F., Jurdic P. (2008). Dual effect of strontium ranelate: Stimulation of osteoblast differentiation and inhibition of osteoclast formation and resorption in vitro. Bone.

[261] Marx D., Rahimnejad Yazdi A., Papini M., Towler M. (2020). A review of the latest insights into the mechanism of action of strontium in bone. Bone Rep..

[262] Jin G., Qin H., Cao H., Qiao Y., Zhao Y., Peng X., Zhang X., Liu X., Chu P.K. (2015). Zn/Ag micro-galvanic couples formed on titanium and osseointegration effects in the presence of *S. aureus*. Biomaterials.

[263] Chen B., You Y., Ma A., Song Y., Jiao J., Song L., Shi E., Zhong X., Li Y., Li C. (2020). Zn-Incorporated TiO_2_ Nanotube Surface Improves Osteogenesis Ability Through Influencing Immunomodulatory Function of Macrophages. Int. J. Nanomed..

[264] O’Connor J.P., Kanjilal D., Teitelbaum M., Lin S.S., Cottrell J.A. (2020). Zinc as a therapeutic agent in bone regeneration. Materials.

[265] Bonaventura P., Benedetti G., Albarède F., Miossec P. (2015). Zinc and its role in immunity and inflammation. Autoimmun. Rev..

[266] Li Y., Liu L., Wan P., Zhai Z., Mao Z., Ouyang Z., Yu D., Sun Q., Tan L., Ren L. (2016). Biodegradable Mg-Cu alloy implants with antibacterial activity for the treatment of osteomyelitis: In vitro and in vivo evaluations. Biomaterials.

[267] Liu R., Tang Y., Zeng L., Zhao Y., Ma Z., Sun Z., Xiang L., Ren L., Yang K. (2018). In vitro and in vivo studies of anti-bacterial copper-bearing titanium alloy for dental application. Dent. Mater..

[268] Liu R., Ma Z., Kolawole S.K., Zeng L., Zhao Y., Ren L., Yang K. (2019). In vitro study on cytocompatibility and osteogenesis ability of Ti–Cu alloy. J. Mater. Sci. Mater. Med..

[269] Li Y., Li B., Song Y., Ma A., Li C., Zhang X., Li H., Zhang Q., Zhang K. (2019). Improved osteoblast adhesion and osseointegration on TiO_2_ nanotubes surface with hydroxyapatite coating. Dent. Mater. J..

[270] Zhang X., Zhang D., Peng Q., Lin J., Wen C. (2019). Biocompatibility of nanoscale hydroxyapatite coating on TiO_2_ nanotubes. Materials.

[271] Çalişkan N., Bayram C., Erdal E., Karahaliloǧlu Z., Denkbaş E.B. (2014). Titania nanotubes with adjustable dimensions for drug reservoir sites and enhanced cell adhesion. Mater. Sci. Eng. C.

[272] Sutrisno L., Hu Y., Shen X., Li M., Luo Z., Dai L., Wang S., Zhong J.L., Cai K. (2018). Fabrication of hyaluronidase-responsive biocompatible multilayers on BMP2 loaded titanium nanotube for the bacterial infection prevention. Mater. Sci. Eng. C.

[273] Zhang X., Zhang Z., Shen G., Zhao J. (2016). Enhanced osteogenic activity and anti-inflammatory properties of Lenti-BMP-2-loaded TiO_2_ nanotube layers fabricated by lyophilization following trehalose addition. Int. J. Nanomed..

[274] Zhang X., Yu Q., Wang Y.A., Zhao J. (2018). Dose reduction of bone morphogenetic protein-2 for bone regeneration using a delivery system based on lyophilization with trehalose. Int. J. Nanomed..

[275] Zhang W., Jin Y., Qian S., Li J., Chang Q., Ye D., Pan H., Zhang M., Cao H., Liu X. (2014). Vacuum extraction enhances rhPDGF-BB immobilization on nanotubes to improve implant osseointegration in ovariectomized rats. Nanomed. Nanotechnol. Biol. Med..

[276] Ma A., Shang H., Song Y., Chen B., You Y., Han W., Zhang X., Zhang W., Li Y., Li C. (2019). Icariin-Functionalized Coating on TiO_2_ Nanotubes Surface to Improve Osteoblast Activity In Vitro and Osteogenesis Ability In Vivo. Coatings.

[277] Zhao X., You L., Wang T., Zhang X., Li Z., Ding L., Li J., Xiao C., Han F., Li B. (2020). Enhanced osseointegration of titanium implants by surface modification with silicon-doped titania nanotubes. Int. J. Nanomed..

[278] Wu F., Wu F., Xu J., Yan R., Yan R., Hu B., Hu B., Li G., Li G., Jin M. (2020). In vitro and in vivo evaluation of antibacterial activity of polyhexamethylene guanidine (PHMG)-loaded TiO_2_ nanotubes. Biomed. Mater..

[279] Somsanith N., Kim Y.K., Jang Y.S., Lee Y.H., Yi H.K., Jang J.H., Kim K.A., Bae T.S., Lee M.H. (2018). Enhancing of osseointegration with propolis-loaded TiO2 nanotubes in rat mandible for dental implants. Materials.

[280] Termaat M.F., Den Boer F., Bakker F., Patka P., Haarman H. (2005). Bone Morphogenetic Proteins. Development and Clinical Efficacy in the Treatment of Fractures and Bone Defects. J. Bone Jt. Surg..

[281] Kanakaris N.K., Giannoudis P. (2008). V Clinical applications of bone morphogenetic proteins: Current evidence. J. Surg. Orthop. Adv..

[282] Caplan A.I., Correa D. (2011). PDGF in bone formation and regeneration: New insights into a novel mechanism involving MSCs. J. Orthop. Res..

[283] Prinsloo P.J.J., Hosking D.J. (2006). Alendronate sodium in the management of osteoporosis. Ther. Clin. Risk Manag..

[284] Zhu Y., Zheng T., Wen L.M., Li R., Zhang Y.B., Bi W.J., Feng X.J., Qi M.C. (2021). Osteogenic capability of strontium and icariin-loaded TiO_2_ nanotube coatings in vitro and in osteoporotic rats. J. Biomater. Appl..

[285] Ma A., You Y., Chen B., Wang W., Liu J., Qi H., Liang Y., Li Y. (2020). Icariin/Aspirin Composite Coating on TiO_2_ Nanotubes Surface Induce Immunomodulatory Effect. Coatings.

[286] Bankova V.S., de Castro S.L., Marcucci M.C. (2000). Propolis: Recent advances in chemistry and plant origin. Apidologie.

[287] Meimandi-Parizi A., Oryan A., Sayahi E., Bigham-Sadegh A. (2018). Propolis extract a new reinforcement material in improving bone healing: An in vivo study. Int. J. Surg..

[288] Wang Z., Wang D., Yang D., Zhen W., Zhang J., Peng S. (2018). The effect of icariin on bone metabolism and its potential clinical application. Osteoporos. Int..

[289] Xie L., Liu N., Xiao Y., Liu Y., Yan C., Wang G., Jing X. (2020). In Vitro and In Vivo Osteogenesis Induced by Icariin and Bone Morphogenetic Protein-2: A Dynamic Observation. Front. Pharmacol..

[290] Zhao L., Wang H., Huo K., Zhang X., Wang W., Zhang Y., Wu Z., Chu P.K. (2013). The osteogenic activity of strontium loaded titania nanotube arrays on titanium substrates. Biomaterials.

[291] Alenezi A., Galli S., Atefyekta S., Andersson M., Wennerberg A. (2019). Osseointegration effects of local release of strontium ranelate from implant surfaces in rats. J. Mater. Sci. Mater. Med..

[292] O’Neill E., Awale G., Daneshmandi L., Umerah O., Lo K.W.-H. (2018). The roles of ions on bone regeneration. Drug Discov. Today.

[293] Xing M., Wang X., Wang E., Gao L., Chang J. (2018). Bone tissue engineering strategy based on the synergistic effects of silicon and strontium ions. Acta Biomater..

[294] Mladenović Ž., Johansson A., Willman B., Shahabi K., Björn E., Ransjö M. (2014). Soluble silica inhibits osteoclast formation and bone resorption in vitro. Acta Biomater..

[295] Losic D., Aw M.S., Santos A., Gulati K., Bariana M. (2015). Titania nanotube arrays for local drug delivery: Recent advances and perspectives. Expert Opin. Drug Deliv..

[296] Losic D. (2021). Advancing of titanium medical implants by surface engineering: Recent progress and challenges. Expert Opin. Drug Deliv..

[297] Vitt A., Sofrata A., Slizen V., Sugars R.V., Gustafsson A., Gudkova E.I., Kazeko L.A., Ramberg P., Buhlin K. (2015). Antimicrobial activity of polyhexamethylene guanidine phosphate in comparison to chlorhexidine using the quantitative suspension method. Ann. Clin. Microbiol. Antimicrob..

[298] Swiontek Brzezinska M., Walczak M., Richert A., Kalwasinska A., Pejchalová M. (2016). The influence of polyhexamethylene guanidine derivatives introduced into polyhydroxybutyrate on biofilm formation and the activity of bacterial enzymes. Appl. Biochem. Microbiol..

